# Structural basis for enhanced infectivity and immune evasion of SARS-CoV-2 variants

**DOI:** 10.1126/science.abi9745

**Published:** 2021-08-06

**Authors:** Yongfei Cai, Jun Zhang, Tianshu Xiao, Christy L. Lavine, Shaun Rawson, Hanqin Peng, Haisun Zhu, Krishna Anand, Pei Tong, Avneesh Gautam, Shen Lu, Sarah M. Sterling, Richard M. Walsh, Sophia Rits-Volloch, Jianming Lu, Duane R. Wesemann, Wei Yang, Michael S. Seaman, Bing Chen

**Affiliations:** 1 Division of Molecular Medicine, Boston Children’s Hospital, 3 Blackfan Street, Boston, MA 02115, USA.; 2 Department of Pediatrics, Harvard Medical School, 3 Blackfan Street, Boston, MA 02115, USA.; 3 Center for Virology and Vaccine Research, Beth Israel Deaconess Medical Center, 330 Brookline Avenue, Boston, MA 02215, USA.; 4 SBGrid Consortium, Harvard Medical School, 250 Longwood Avenue, Boston, MA 02115, USA.; 5 The Harvard Cryo-EM Center for Structural Biology, Harvard Medical School, 250 Longwood Avenue, Boston, MA 02115, USA.; 6 Department of Biological Chemistry and Molecular Pharmacology, Blavatnik Institute, Harvard Medical School, 240 Longwood Avenue, Boston, MA 02115, USA.; 7 Institute for Protein Innovation, Harvard Institutes of Medicine, 4 Blackfan Circle, Boston, MA 02115, USA.; 8 Division of Allergy and Immunology and Division of Genetics, Department of Medicine, Brigham and Women’s Hospital, Harvard Medical School, 75 Francis Street, Boston, MA 02115, USA.; 9 Codex BioSolutions, Inc., 401 Professional Drive, Gaithersburg, MD 20879, USA.; 10 Department of Biochemistry and Molecular and Cellular Biology, Georgetown University School of Medicine, 3900 Reservoir Road, NW, Washington, DC 20057, USA.

## Abstract

Several fast-spreading variants of severe acute respiratory syndrome coronavirus 2 (SARS-CoV-2) have become the dominant circulating strains in the COVID-19 pandemic. We report here cryo–electron microscopy structures of the full-length spike (S) trimers of the B.1.1.7 and B.1.351 variants, as well as their biochemical and antigenic properties. Amino acid substitutions in the B.1.1.7 protein increase both the accessibility of its receptor binding domain and the binding affinity for receptor angiotensin-converting enzyme 2 (ACE2). The enhanced receptor engagement may account for the increased transmissibility. The B.1.351 variant has evolved to reshape antigenic surfaces of the major neutralizing sites on the S protein, making it resistant to some potent neutralizing antibodies. These findings provide structural details on how SARS-CoV-2 has evolved to enhance viral fitness and immune evasion.

## SARS-CoV-2 from alpha to epsilon

As battles to contain the COVID-19 pandemic continue, attention is focused on emerging variants of the severe acute respiratory syndrome coronavirus 2 (SARS-CoV-2) virus that have been deemed variants of concern because they are resistant to antibodies elicited by infection or vaccination or they increase transmissibility or disease severity. Three papers used functional and structural studies to explore how mutations in the viral spike protein affect its ability to infect host cells and to evade host immunity. Gobeil *et al*. looked at a variant spike protein involved in transmission between minks and humans, as well as the B1.1.7 (alpha), B.1.351 (beta), and P1 (gamma) spike variants; Cai *et al*. focused on the alpha and beta variants; and McCallum *et al*. discuss the properties of the spike protein from the B1.1.427/B.1.429 (epsilon) variant. Together, these papers show a balance among mutations that enhance stability, those that increase binding to the human receptor ACE2, and those that confer resistance to neutralizing antibodies. —VV

## Teaser

How SARS-CoV-2 variants gain enhanced infectivity and evade host immune responses.

The COVID-19 pandemic, caused by severe acute respiratory syndrome coronavirus 2 (SARS-CoV-2) (*1*), has led to millions of lives lost and devastating socioeconomic disruptions worldwide. Although the mutation rate of the coronavirus is relatively low because of the proofreading activity of its replication machinery (*2*), several variants of concern have emerged—including the B.1.1.7 lineage first identified in the United Kingdom, the B.1.351 lineage in South Africa, and the B.1.1.28 lineage in Brazil—within a period of several months (*3*–*5*). These variants not only appear to spread more efficiently than the virus from the initial outbreak [i.e., the strain Wuhan-Hu-1; (*1*)] but also may be more resistant to immunity elicited by the Wuhan-Hu-1 strain after either natural infection or vaccination (*6*–*8*). The B.1.1.7 variant is of particular concern because it has been reported to be more deadly (*9*, *10*). Thus, understanding the underlying mechanisms of the increased transmissibility, risk of mortality, and immune resistance of new variants may facilitate development of intervention strategies to control the crisis.

SARS-CoV-2 is an enveloped positive-stranded RNA virus that depends on fusion of viral and target cell membranes to enter a host cell. This first key step of infection is catalyzed by the virus-encoded trimeric spike (S) protein, which is also a major surface antigen and thus an important target for development of diagnostics, vaccines, and therapeutics. The S protein is synthesized as a single-chain precursor and is subsequently cleaved by a furin-like protease into the receptor-binding fragment S1 and the fusion fragment S2 [fig. S1 and (*11*)]. Binding of the viral receptor angiotensin-converting enzyme 2 (ACE2) on the host cell surface to the receptor-binding domain (RBD) of S1, together with a second proteolytic cleavage by another cellular protease in S2 [S2′ site; fig. S1 and (*12*)], induces dissociation of S1 and irreversible refolding of S2 into a postfusion structure, ultimately leading to membrane fusion (*13*, *14*). In the prefusion conformation, S1 folds into four domains—NTD (N-terminal domain), RBD, and two CTDs (C-terminal domains)—that wrap around the prefusion S2 structure. The RBD can adopt two distinct conformations: “up” for a receptor-accessible state and “down” for a receptor-inaccessible state (*15*). Rapid progress in structural biology of the S protein has advanced our knowledge of the SARS-CoV-2 entry process (*15*–*28*). We have previously identified two structural elements, the FPPR (fusion peptide proximal region) and the 630 loop, which appear to modulate the S protein stability as well as the RBD conformation and thus the receptor accessibility (*22*, *28*).

The S protein is the basis of almost all the first-generation COVID-19 vaccines, which were developed using the Wuhan-Hu-1 sequence (*29*, *30*). Several have received emergency use authorization by various regulatory agencies throughout the world because of their impressive protective efficacy and minimal side effects (*31*, *32*). These vaccines appear to have somewhat lower efficacy against the B.1.351 variant than against its parental strain (*6*–*8*, *33*), and this variant became completely resistant to many convalescent serum samples in vitro (*8*). How to address genetic diversity has therefore become a high priority for developing next-generation vaccines. In this study, we have characterized the full-length S proteins from the B.1.1.7 and B.1.351 variants and determined their structures by cryo–electron microscopy (cryo-EM), providing a structural basis for understanding the molecular mechanisms of the enhanced infectivity of B.1.1.7 and the immune evasion of B.1.351.

## Biochemical and antigenic properties of the intact S proteins from the new variants

To characterize the full-length S proteins with the sequences derived from natural isolates of the B.1.1.7 (hCoV-19/England/MILK-C504CD/2020) and B.1.351 (hCoV-19/South Africa/KRISP-EC-MDSH925100/2020) variants (fig. S1), we first transfected human embryonic kidney (HEK) 293 cells with the respective expression constructs and compared their membrane fusion activities with those of the full-length S constructs of their parental strains [Wuhan-Hu-1: D614 (Asp at position 614) and its early D614G variant: G614 (Asp-to-Gly mutation at position 614) (*34*)]. All S proteins expressed at comparable levels (fig. S2A), and the cells producing these S proteins fused efficiently with ACE2-expressing cells (fig. S2B). Consistent with our previous findings (*22*, *28*), the G614 and B.1.351 variant S constructs showed slightly higher fusion activity than the D614 and B.1.1.7 variants, but the differences diminished when the transfection level increased.

To produce the full-length S proteins, we added a C-terminal strep-tag to the B.1.1.7 and B.1.351 S (fig. S3A) and expressed and purified these proteins under the conditions established for producing the D614 and G614 S trimers (*22*, *28*). The B.1.1.7 protein eluted in three distinct peaks, representing the prefusion S trimer, postfusion S2 trimer, and dissociated S1 monomer, respectively (*22*), consistent with Coomassie-stained SDS–polyacrylamide gel electrophoresis (SDS-PAGE) analysis (fig. S3B). Nonetheless, the prefusion trimer was the predominant form, accounting for >70% of the total protein, indicating that this trimer is more stable than D614, where the prefusion trimer was only <25%. Like the G614 trimer (*28*), B.1.351 protein eluted in a single major peak, corresponding to the prefusion S trimer (fig. S3B), with no obvious peaks for dissociated S1 and S2. SDS-PAGE analysis showed that the prefusion trimer peaks contained primarily the cleaved S1/S2 complex for both the proteins, with the cleavage level moderately higher for B.1.351 than for B.1.1.7. These results indicate that the B.1.351 and G614 S proteins have almost identical biochemical properties, whereas the B.1.1.7 trimer is slightly less stable.

**Fig. 1.**
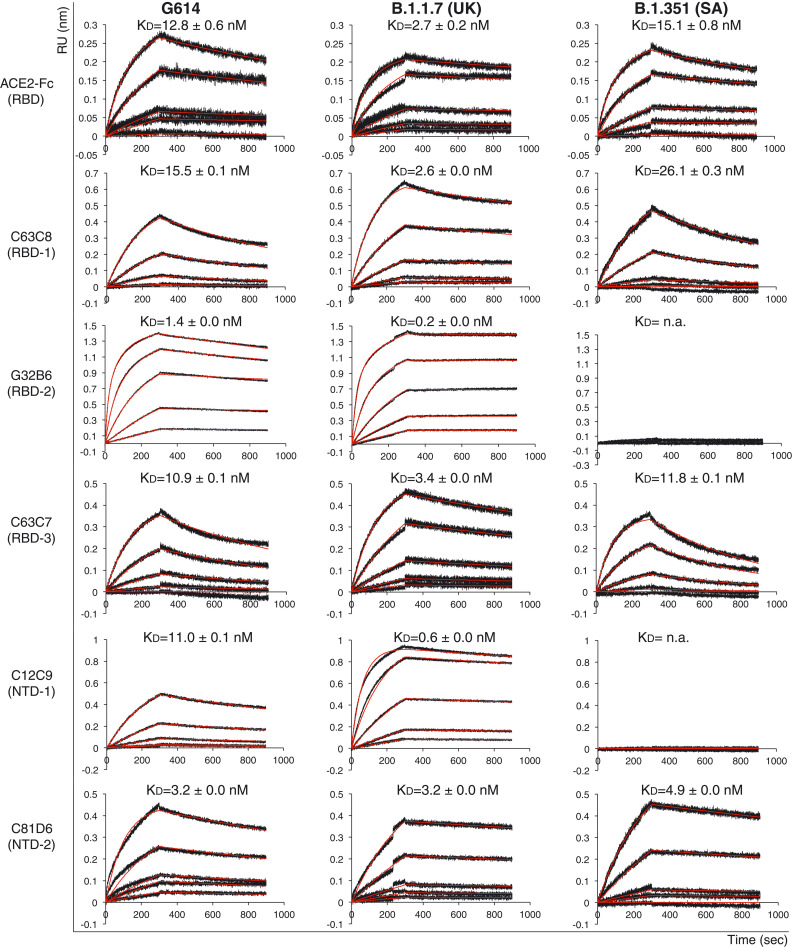
**Antigenic properties of the purified full-length SARS-CoV-2 S proteins.** BLI analysis of the association of prefusion S trimers from the G614 “parent” strain and the B.1.1.7 and B.1.351 variants derived from it with soluble ACE2 constructs and with a panel of antibodies representing five epitopic regions on the RBD and NTD [see fig. S4A and (*35*)]. For ACE2 binding, purified S proteins were immobilized to AR2G (Amine Reactive 2nd Generation) biosensors and dipped into the wells containing ACE2 at different concentrations. For antibody binding, various antibodies were immobilized to AHC (Anti-human IgG Fc Capture) biosensors and dipped into the wells containing each purified S protein at different concentrations. Binding kinetics were evaluated using a 1:1 Langmuir model except for dimeric ACE2 and antibody G32B6 targeting the RBD-2, which were analyzed by a bivalent binding model. The sensorgrams are in black and the fits in red. Binding constants are also summarized here and in table S1. All experiments were repeated at least twice with essentially identical results. K_D_, dissociation constant; n.a., not available; RU, response unit.

To assess antigenic properties of the prefusion variant S trimers, we measured their binding to soluble ACE2 and S-directed monoclonal antibodies isolated from COVID-19 convalescent individuals by bio-layer interferometry (BLI). These antibodies target various epitopic regions on the S trimer, as defined by clusters of competing antibodies and designated RBD-1, RBD-2, RBD-3, NTD-1, NTD-2, and S2 [fig. S4A; (*35*)]. All but the last two clusters contain neutralizing antibodies. The B.1.1.7 variant bound stronger to the receptor than did its G614 parent, regardless of the ACE2 oligomeric state (Fig. 1, fig. S4B, and table S1). The B.1.351 trimer had higher affinity for monomeric ACE2, but slightly lower affinity for dimeric ACE2, than the G614 trimer. In both cases, affinity for ACE2 of the B.1.351 protein was lower than that of the B.1.1.7 variant. These results suggest that the mutation in the RBD of the B.1.1.7 variant [N501Y (Asn^501^→Tyr)] enhances receptor recognition, whereas the additional mutations in the B.1.351 variant [K417N (Lys^417^→Asn) and E484K (Glu^484^→Lys)] reduce ACE2 affinity to a level close to that of the G614 protein, consistent with the previous data (*36*, *37*). All selected monoclonal antibodies bound G614 S with reasonable affinities, and the B.1.1.7 variant showed a similar pattern but with substantially stronger binding to almost all the antibodies (Fig. 1, fig. S4B, and table S1). By contrast, the B.1.351 variant completely lost binding to the two RBD-2 antibodies, G32B6 and C12A2, as well as to the two NTD-1 antibodies, C12C9 and C83B6, whereas the affinities for the rest of the antibodies were the same as those of the G614 trimer. The BLI data were also consistent with the binding results with the membrane-bound S trimers measured by flow cytometry (fig. S5).

We next assessed the neutralization potency of the antibodies and the trimeric ACE2 construct in blocking infection of these variants in an HIV-based pseudovirus assay. For most antibodies, the neutralization potency correlated with their binding affinity for the membrane-bound or purified S proteins (fig. S6 and table S2). C81D6 and C163E6 recognize two non-neutralizing epitopes, located in the NTD and S2, respectively, and they did not neutralize any of the pseudoviruses. The B.1.1.7 virus is the most sensitive to the trimeric ACE2 and the RBD-up–targeting C63C7, suggesting that the B.1.1.7 trimer may prefer the RBD-up conformation. Thus, the detergent-solubilized S proteins adopt a physiologically relevant conformation, and mutations in B.1.351 have a greater impact on the antibody sensitivity of the virus than those in B.1.1.7.

## Structures of the full-length S trimers from the B.1.1.7 and B.1.351 variants

We determined the cryo-EM structures of the full-length S trimers with the unmodified sequences of the B.1.1.7 and B.1.351 variants. Cryo-EM images were acquired on a Titan Krios electron microscope equipped with a Gatan K3 direct electron detector. We used RELION (*38*) for particle picking, two-dimensional (2D) classification, 3D classification, and refinement (figs. S7 to S10), and cryoSPARC (*39*) for validation. 3D classification identified five distinct classes for the B.1.1.7 S trimer—representing one closed prefusion conformation, three one-RBD-up conformations, and one two-RBD-up conformation—and two different classes for the B.1.351 trimer, representing a closed conformation and a one-RBD-up conformation. These structures were refined to 2.9- to 4.3-Å resolution (figs. S7 to S10 and table S3).

The overall architectures of the full-length variant S proteins are very similar to that of the G614 S trimer in the corresponding conformation [figs. S11 and S12; (*28*)]. In the closed, three-RBD-down structure, the four domains of S1—NTD, RBD, CTD1, and CTD2—wrap around the prefusion S2 trimer. In the one-RBD-up conformation, the RBD position has no effect on the central core region of S2, but two NTDs, the immediately adjacent one and the one from the same protomer, shift away from the threefold axis and open up the trimer. The furin cleavage site at the S1/S2 boundary (residues 682 to 685) in these structures remains disordered, and the structures therefore cannot explain the difference in the cleavage level between the B.1.1.7 and B.1.351 trimers; the position of a substitution [P681H (Pro^681^→His)] in the B.1.1.7 S (fig. S1) close to the cleavage site is likewise not visible. A small class of particles in the two-RBD-up conformation was present only with the B.1.1.7 trimer (fig. S11), possibly because B.1.1.7 S1 is less likely to dissociate.

**Fig. 2.**
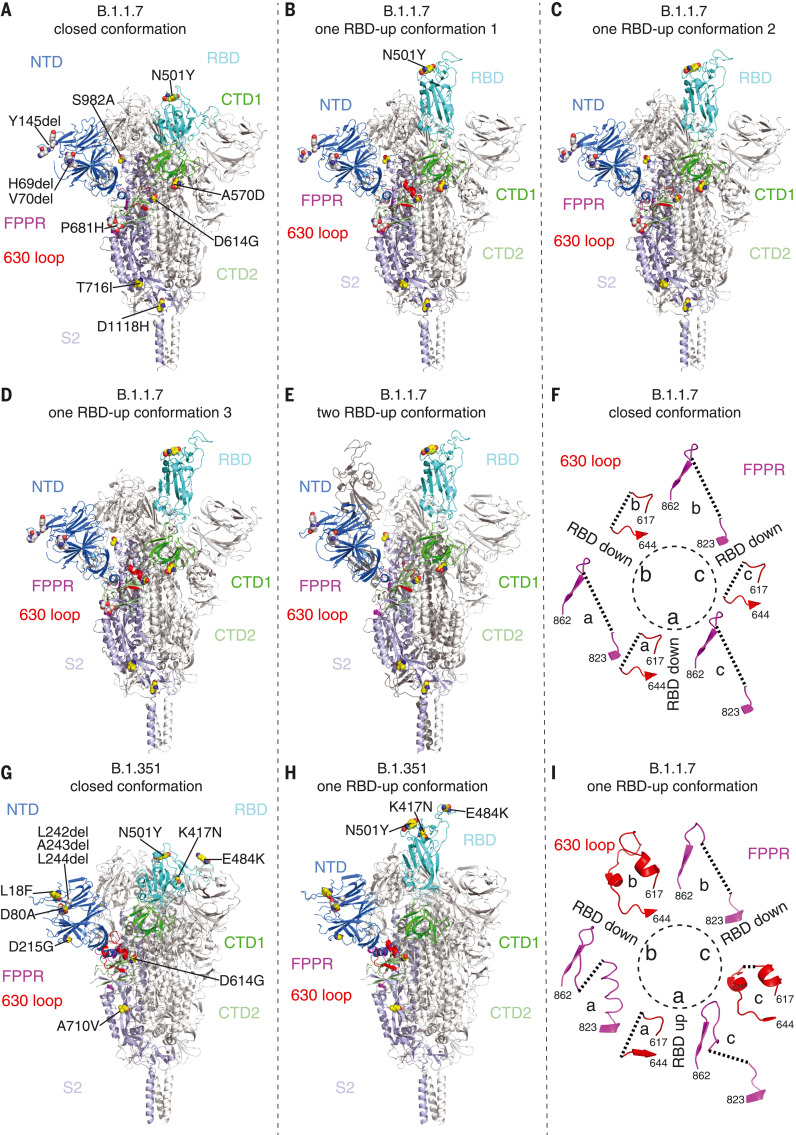
Cryo-EM structures of the full-length SARS-CoV-2 S proteins from the B.1.1.7 and B.1.351 variants. (**A** to **E**) The structures of the closed prefusion conformation (A), three one-RBD-up conformations [(B) to (D)], and a two-RBD-up conformation (E) of the B.1.1.7 S trimer are shown in ribbon diagram, with one protomer colored as NTD in blue, RBD in cyan, CTD1 in green, CTD2 in light green, S2 in light blue, the 630 loop in red, and the FPPR in magenta. (**G** and **H**) The structures of the closed prefusion conformation (G) and one-RBD-up conformation (H) of the B.1.351 S trimer are shown in ribbon diagram with the same color scheme as in (A) to (E). All mutations in the new variants, as compared with the original virus (D614), are highlighted as sphere models. (**F** and **I**) Structures, in the B.1.1.7 trimer, of segments (residues 617 to 644) containing the 630 loop (red) and segments (residues 823 to 862) containing the FPPR (magenta) from each of the three protomers (a, b, and c). The position of each RBD is indicated. Dashed lines indicate gaps in the chain trace (disordered loops).

For the B.1.1.7 S trimer, most particles used for refinement were in the RBD-up conformation (Fig. 2, A to E). We have proposed that the FPPR (residues 828 to 853) and 630 loop (residues 620 to 640) modulate the stability and fusogenic structural rearrangements of the S protein (*22*, *28*). In the closed conformation of the B.1.1.7 trimer, all three FPPR and three 630 loops are disordered (Fig. 2F), which otherwise would help clamp down the RBDs. This explains why the B.1.1.7 trimer is more likely than its parental G614 variant to populate the RBD-up conformation, because the FPPRs and 630 loops are structured in the G614 trimer (*28*). In the one-RBD-up conformation, one 630 loop on the opposite side of the up RBD becomes fully structured, inserting between neighboring NTD and CTDs in the same configuration found in the G614 trimer (*28*). The second 630 loop is partially ordered, whereas the third one remains disordered. A similar pattern is found for three FPPRs, although the structured FPPR adopts a conformation distinct from the one seen in our previous structures of the full-length S proteins (*22*, *28*). Overall, the arrangement of these structural elements appears to stabilize the cleaved S trimer and to prevent the premature S1 dissociation in the one-RBD-up conformation. The three one-RBD-up structures differ only by the degree to which the up RBD and the adjacent NTD of its neighboring protomer shift away from the central threefold axis (fig. S13A). We have suggested that the two-RBD-up conformation might be unstable (*22*, *28*), leading to S1 dissociation and irreversible S2 refolding. If this suggestion is valid, the small class of the two-RBD-up particles probably contains mainly uncleaved S trimers.

The two classes for the B.1.351 S trimer represent the closed prefusion and one-RBD-up states, respectively (Fig. 2, G and H). The configurations of the FPPR and 630 loop closely follow the distribution seen in the G614 trimer: All are structured in the RBD-down conformation, whereas only the FPPR and 630-loop pair is ordered in the one-RBD-up conformation [fig. S12; (*28*)]. These observations are consistent with the similar biochemical stabilities of the B.1.351 and G614 S trimers [fig. S3; (*28*)].

## Structural consequences of mutations in the B.1.1.7 variant

**Fig. 3.**
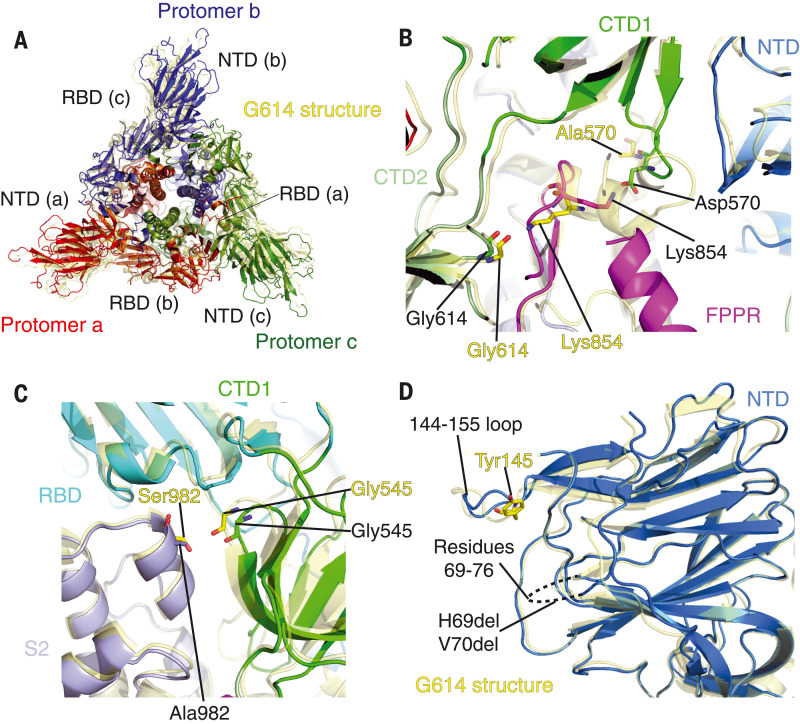
**Structural impact of the mutations in the B.1.1.7 S.** (**A**) Top views of superposition of the structure of the B.1.1.7 S trimer in ribbon representation, with the structure of the prefusion trimer of the G614 S [Protein Data Bank (PDB) ID: 7KRQ] shown in yellow. The NTD and RBD of each protomer are indicated. (**B**) A close-up view of the region near the A570D mutation with superposition of the B.1.1.7 trimer structure (one-RBD-up) in green (CTD1) and magenta (FPPR) and the G614 trimer (closed) in yellow. Residues Ala^570^, Asp^570^, two Gly^614^, and two Lys^854^ from both structures are shown in stick model. (**C**) A view of the region near the S982A mutation with superposition of the B.1.1.7 trimer structure (closed) in green (CTD1) and magenta (FPPR) and the G614 trimer (closed) in yellow. (**D**) Superposition of the NTD structure of the B.1.1.7 S trimer in blue with the NTD of the G614 S trimer in yellow. Locations of Tyr^145^ and the disordered loop containing residues 69 to 76 are indicated.

We superposed the structures of the B.1.1.7 trimer onto the G614 trimer in the closed conformation, aligning them by the S2 structure (Fig. 3A). An outward rotation of all three S1 subunits in B.1.1.7 leads to a slightly more open conformation. This rotation in B.1.1.7 widens the gap between the NTD and the CTDs of the same protomer (fig. S13B). In the G614 trimer, this gap accommodates the ordered 630 loop that reinforces CTD2 and prevents S1 shedding (*28*). The widened gap in the variant loosens the grip on the 630 loop, accounting for the absence of ordered features in this part of the B.1.1.7 map. There are two mutations that may be responsible for these structural differences. First, Ala^570^ in CTD1 packs against one side of the FPPR in the G614 trimer (Fig. 3B). The A570D (Ala^570^→Asp) mutation, with a larger side chain, may weaken the packing and destabilize the FPPR. Moreover, in the one-RBD-up conformation of the B.1.1.7 S, in which the FPPR is at least partially structured, Lys^854^, which in the G614 trimer probably forms a hydrogen bond with the main chain carbonyl group of Gly^614^, flips back in B.1.1.7 to form a salt bridge with the mutant Asp^570^. Second, S982A (Ser^982^→Ala) eliminates a hydrogen bond between the central helices of S2 and the carbonyl group of Gly^545^ in CTD1 (Fig. 3C). These two mutations together allow an outward movement of CTD1 by more than 3 Å (fig. S13B), thereby affecting the conformation of the FPPR and 630 loops. In the one-RBD-up conformation, the NTD and CTDs on the opposite side of the up RBD move closer together, narrowing the gap between them and stabilizing the structured 630 loop.

Other mutations in B.1.1.7 cluster in the NTD, including deletions of His^69^, Val^70^, and Tyr^145^ (Fig. 3D). The first two residues are in a disordered loop in all these S structures, and the structural impact of their deletion is unclear. Tyr^145^ is also near a loop (residues 144 to 155), and its deletion apparently causes only some local changes of the loop. The absence of structural changes in the B.1.1.7 NTD is consistent with the absence of effects on its sensitivity to the various NTD-directed antibodies (*35*). Additional mutations [N501Y, T716I (Thr^716^→Ile), and D1118H (Asp^1118^→His)] caused minimal local changes (fig. S14, A to C).

## Structural impact of the mutations in the B.1.351 variant

**Fig. 4.**
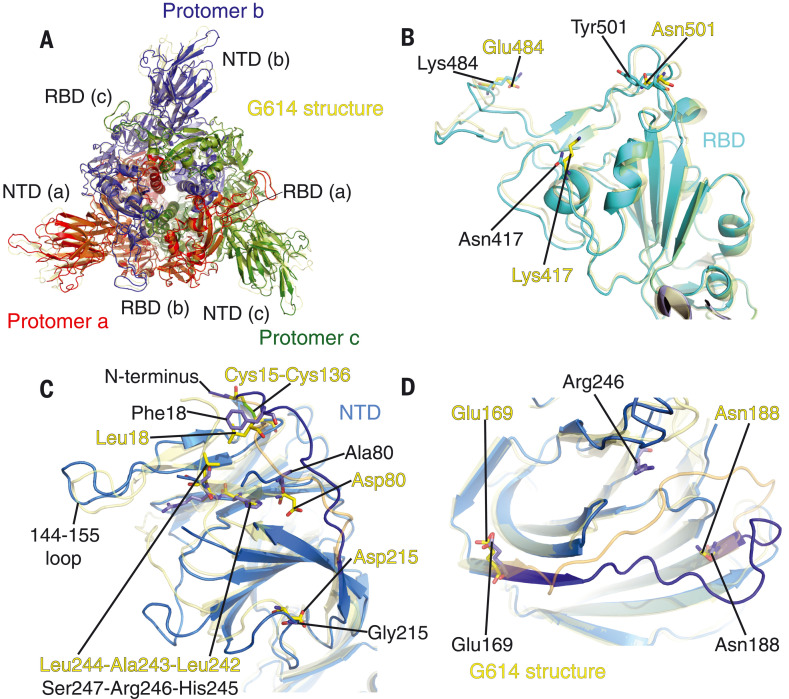
**Structural impact of the mutations in the B.1.351 S.** (**A**) Top views of superposition of the structure of the B.1.351 S trimer in ribbon representation, with the structure of the prefusion trimer of the G614 S (PDB ID: 7KRQ) shown in yellow. The NTD and RBD of each protomer are indicated. (**B**) Superposition of the RBD structure of the B.1.351 S trimer in blue with the RBD of the G614 S trimer in yellow. Locations of mutations K417N, E484K, and N501Y are indicated, and these residues are shown in stick model. (**C**) A view of the NTDs from superposition of the structure of the B.1.351 S trimer in blue and the G614 S in yellow. Locations of mutations L18F, D80A, and D215G; the disulfide bond between Cys^15^ and Cys^136^; and replacement of Leu^242^-Ala^243^-Leu^244^ by His^245^-Arg^246^-Ser^247^ are indicated, and the residues are shown in stick model. (**D**) Superposition of the NTD structure of the B.1.351 S trimer in blue with the NTD of the G614 S trimer in yellow. Displacement of the segment 169 to 188 and the location of Arg^246^ in the B.1.351 structure are indicated.

The overall structures of the B.1.351 and G614 trimers were essentially the same for the corresponding states, except for some loop regions in the NTD (Fig. 4A and fig. S15). Three mutations—K417N, E484K, and N501Y—at the ACE2 binding site do not produce any major structural rearrangements (Fig. 4B). The most notable differences are in the NTD, which contains three point mutations [L18F(Leu^18^→Phe), D80A (Asp^80^→Ala), and D215G (Asp^215^→Gly)] and a three-residue deletion (L242del, A243del, and L244del). The L18F and D80A changes lead to reconfiguration of the N-terminal segment despite the disulfide between Cys^15^ and Cys^136^ that partly anchors the N-terminal peptide (Fig. 4C). D215G appears to have the least structural impact because Asp^215^ is a solvent-exposed residue that may compensate for the surface charge from the neighboring, well-exposed Arg^214^.

The most consequential changes are probably from the triple-residue deletion, because these nonpolar residues, located on the edge of the NTD core structure formed by four stacking β sheets, are replaced with polar residues His^245^-Arg^246^-Ser^247^. This replacement causes a shift of the nearby loop (residues 144 to 155) and must also reconfigure the adjacent disordered loop (residues 246 to 260), both of which form part of the NTD neutralizing epitopes (*40*). Furthermore, Arg^246^ is pointing toward the side chain of Arg^102^ near the segment 172 to 188, forcing this loop to rearrange. As shown in Fig. 4D, the 172-to-188 segment wraps around the edge of the NTD core, packing against Leu^242^-Ala^243^-Leu^244^ at the edge of the β sheet in the G614 trimer. The triple-residue deletion rearranges the 172-to-188 segment with a movement up to 17 Å (Leu^180^). By substantially altering the conformational preferences of this component of the molecular surface, these mutations likely affect binding of any antibody that has part of its footprint in this region. The additional mutation A701V (Ala^701^→Val) is located in the surface-exposed region of S2 and caused minimal structural changes (fig. S14D).

## Discussion

Transmissibility and immune evasion are independent selective forces driving emergence of viral genetic diversity. The changes of most concern in the SARS-CoV-2 S protein would be those that simultaneously enhance transmission, augment disease severity, and evade immune recognition in previously exposed hosts. Our data suggest that the most problematic combination of such mutations is not yet present in the existing variants examined here.

In the B.1.1.7 virus, mutations A570D and S982A lead to an outward shift of the CTD1, thereby relaxing the FPPR and 630 loop, which help retain the RBD in its “down” position in the parental strain. The mutations increase the frequency with which the S trimer samples the RBD-up conformation, allowing B.1.1.7 to better present the receptor binding motif (RBM) to ACE2 on the host cells. Once one RBD flips up, the fully or partially ordered 630 loops of the neighboring protomers stabilize the CTD2, which folds together with the N-terminal segment of S2, and thus prevent the premature S1 dissociation. N501Y in the ACE2 binding site of the RBD also increases the affinity of that domain for the receptor, probably because of the hydrophobic interaction of Tyr^501^ with Tyr^41^ of ACE2 (*36*) and a possible cation-π interaction with ACE2 Lys^353^ (fig. S16). The combination of enhanced RBM presentation and additional local interactions might allow the B.1.1.7 virus to infect cell types with lower ACE2 levels than those of the nasal and bronchial epithelial cells that the virus typically infects; an expanded cell tropism could account for the increased risk of mortality in patients infected with this variant (*9*, *10*). The mutations in B.1.1.7 caused no major structural rearrangements in the RBD and NTD, consistent with minimal changes in the sensitivity of the B.1.1.7 variant to the potently neutralizing antibodies [tables S1 and S2; (*33*)].

In the B.1.351 virus, the S protein largely retains the structure of the G614 trimer with almost identical biochemical stability. N501Y, K417N, and E484K in the RBD have not caused major structural changes, but the loss of salt bridges between Lys^417^ and ACE2 Asp^30^ and Glu^484^ and ACE2 Lys^31^ mitigates the increased receptor affinity imparted by N501Y (fig. S16). K417N and E484K probably lead to loss of binding and neutralization by antibodies that target the RBD-2 epitopes (fig. S4A). The accompanying mutations in the NTD remodel the antigenic surface and greatly reduce the potency of neutralizing antibodies against NTD-1 epitopes. The B.1.351 variant was probably selected under a certain level of immune pressure, because it altered two major neutralizing sites on the S trimer simultaneously with only a slight compromise in its ability to engage a host cell.

The global range of SARS-CoV-2 and the daily vast number of replication events make emergence of new variants inevitable and substantially increases the viral genetic diversity. In many cases, antibody resistance may compromise viral fitness, as in the B.1.351 variant, which resists neutralization by RBD-directed antibodies but also loses the enhanced affinity and transmissibility imparted by N501Y, as a consequence of the immune-escape mutations. It is also possible to combine immune evasion and virulence through continuous viral evolution, such as a B.1.1.7 variant that contains the E484K mutation (B.1.1.7+E484K) (*41*). Such a combination will bring greater challenges for vaccine development compared with the beginning of the pandemic. If SARS-CoV-2 becomes seasonal, innovative strategies already developed against other human pathogens—such HIV-1, hepatitis C virus, and influenza virus—may be applicable to on-going control of the COVID-19 pandemic. The B.1.351 S trimer, which has superior biochemical stability and new epitopes, should be an excellent starting point for developing next-generation vaccines designed to elicit broadly neutralizing antibody responses.

## Supplementary Material

Figures and Tables

Checklist

## Supplementary Materials

science.sciencemag.org/content/373/6555/642/suppl/DC1
Materials and Methods Figs. S1 to S16 Tables S1 to S3 References (*42*–*51*) MDAR Reproducibility Checklist

## Appendix

View/request a protocol for this paper from *Bio-protocol*.
