# Supplementary material for: Structural basis for enhanced infectivity and immune evasion of SARS-CoV-2 variants
Source: Science. 2021 Jun 24;373(6555):642–8. doi: 10.1126/science.abi9745 (PMC9245151; doi:10.1126/science.abi9745)
Supplement: Figures and Tables [file abi9745_cai_sm.pdf]

science.sciencemag.org/cgi/content/full/science.abi9745/DC1

Supplementary Materials for

Structural basis for enhanced infectivity and immune evasion of SARS-CoV-2 variants

Yongfei Cai†, Jun Zhang†, Tianshu Xiao†, Christy L. Lavine, Shaun Rawson, Hanqin Peng, Haisun Zhu, Krishna Anand, Pei Tong, Avneesh Gautam, Shen Lu, Sarah M. Sterling, Richard M. Walsh Jr., Sophia Rits-Volloch, Jianming Lu, Duane R. Wesemann, Wei Yang, Michael S. Seaman, Bing Chen*

†These authors contributed equally to this work.
*Corresponding author. Email: bchen@crystal.harvard.edu

Published 24 June 2021 on *Science* First Release
DOI: 10.1126/science.abi9745

This PDF file includes:

Materials and Methods
Figs. S1 to S16
Tables S1 to S3
References

Other Supplementary Material for this manuscript includes the following:
(available at science.sciencemag.org/cgi/content/full/science.abi9745/DC1)

MDAR Reproducibility Checklist (.pdf)

Materials and Methods

Expression constructs

Genes of full-length spike (S) protein from hCoV-19/England/MILK-C504CD/2020 (GISAID accession ID: EPI_ISL_736724) and hCoV-19/South Africa/KRISP-EC-MDSH925100/2020 (GISAID accession ID: EPI_ISL_736980) were synthesized by GENEWIZ (South Plainfield, NJ). The S genes were fused with a C-terminal twin Strep tag [$(GGGGS)_2$WSHPQFEK$(GGGGS)_2$WSHPQFEK)] and cloned into a mammalian cell expression vector pCMV-IRES-puro (Codex BioSolutions, Inc, Gaithersburg, MD).

Expression and purification of recombinant proteins

Expression and purification of the full-length S proteins were carried out as previously described (*22*). Briefly, expi293F cells (ThermoFisher Scientific, Waltham, MA) were transiently transfected with the S protein expression constructs. To purify the S protein, the transfected cells were lysed in a buffer containing Buffer A (100 mM Tris-HCl, pH 8.0, 150 mM NaCl, 1 mM EDTA) and 1% (w/v) n-dodecyl-β-D-maltopyranoside (DDM) (Anatrace, Inc. Maumee, OH), EDTA-free complete protease inhibitor cocktail (Roche, Basel, Switzerland), and incubated at 4°C for one hour. After a clarifying spin, the supernatant was loaded on a strep-tactin column equilibrated with the lysis buffer. The column was then washed with 50 column volumes of Buffer A and 0.3% DDM, followed by additional washes with 50 column volumes of Buffer A and 0.1% DDM, and with 50 column volumes of Buffer A and 0.02% DDM. The S protein was eluted by Buffer A containing 0.02% DDM and 5 mM desthiobiotin. The protein was further purified by gel filtration chromatography on a Superose 6 10/300 column (GE Healthcare, Chicago, IL) in a buffer containing 25 mM Tris-HCl, pH 7.5, 150 mM NaCl, 0.02% DDM.

The monomeric ACE2 or dimeric ACE2 proteins were produced as described (*42*). Briefly, Expi293F cells transfected with monomeric ACE2 or dimeric ACE2 expression construct and the supernatant of the cell culture was collected. The monomeric ACE2 protein was purified by affinity chromatography using Ni Sepharose excel (Cytiva Life Sciences, Marlborough, MA), followed by gel filtration chromatography. The dimeric ACE2 protein was purified by GammaBind Plus Sepharose beads (GE Healthcare), followed gel filtration chromatography on a

Superdex 200 Increase 10/300 GL column. All the monoclonal antibodies were produced as described (*35*).

Western blot

Western blot was performed using an anti-SARS-COV-2 S antibody following a protocol described previously (*43*). Briefly, full-length S protein samples were prepared from cell pellets and resolved in 4-15% Mini-Protean TGX gel (Bio-Rad, Hercules, CA) and transferred onto PVDF membranes. Membranes were blocked with 5% skimmed milk in PBS for 1 hour and incubated a SARS-CoV-2 (2019-nCoV) Spike RBD Antibody (Sino Biological Inc., Beijing, China, Cat: 40592-T62) for another hour at room temperature. Alkaline phosphatase conjugated anti-Rabbit IgG (1:5000) (Sigma-Aldrich, St. Louis, MO) was used as a secondary antibody. Proteins were visualized using one-step NBT/BCIP substrates (Promega, Madison, WI).

Cell-cell fusion assay

The cell-cell fusion assay, based on the α-complementation of E. coli β-galactosidase, was conducted to quantify the fusion activity mediated by SARS-CoV2 S protein, as described (*22*). Briefly, various amount of the full-length SARS-CoV2 (D614, G614, UK and South Africa) S construct (0.025-10 μg) and the α fragment of E. coli β-galactosidase construct (10 μg), or the full-length ACE2 construct (10 μg) together with the ω fragment of E. coli β-galactosidase construct (10 μg), were transfected to HEK293T cells using Polyethylenimine (PEI) (80 μg). After a 24-hour incubation at 37°C, the cells were detached using PBS buffer with 5mM EDTA and resuspended in complete DMEM medium. 50 μl S-expressing cells ($1.0x10^6$ cells/ml) were mixed with 50 μl ACE2-expressing cells ($1.0x10^6$ cells/ml) to allow the cell-cell fusion proceed at 37 °C for 2 hours. Cell-cell fusion activity was quantified using a chemiluminescent assay system, Gal-Screen (Applied Biosystems, Foster City, CA), following the standard protocol recommended by the manufacturer. The substrate was added to the mixture of the cells and allowed to react for 90 minutes in dark at room temperature. The luminescence signal was recorded with a Synergy Neo plate reader (Biotek, Winooski, VT).

Binding assay by bio-layer interferometry (BLI)

Binding of monomeric or dimeric ACE2 to the full-length Spike protein of each variant was measured using an Octet RED384 system (ForteBio, Fremont, CA), following the protocol described previously (*42*). Briefly, the full-length S protein was immobilized to Amine Reactive 2nd Generation (AR2G) biosensors (ForteBio, Fremont, CA) and dipped in the wells containing the ACE2 protein at various concentrations (6.30-510 nM for monomeric ACE2; 1.05-85 nM for dimeric ACE2) for 5 minutes, followed with 10 minutes in the running buffer (PBS, 0.02% Tween 20, 2 mg/ml BSA) to determine the dissociation rate. To measure the binding of the full-length Spike protein to monoclonal antibodies, the antibody was immobilized to anti-human IgG Fc Capture (AHC) biosensor (ForteBio, Fremont, CA) following a protocol recommended by the manufacturer. The full-length Spike protein was diluted using the running buffer (PBS, 0.02% Tween 20, 0.02% DDM, 2 mg/ml BSA) to various concentrations (0.93-75 nM) and transferred to 96-well plate. The sensors were dipped in the wells containing the Spike protein solutions for 5 minutes to measure the association rate, followed with 10 minutes in the wells of the running buffer to measure the dissociation rate. Control sensors with no Spike protein or antibody were also dipped in the ACE2 or Spike protein solutions and the running buffer as references. Recorded sensorgrams with background subtracted from the references were analyzed using the software Octet Data Analysis HT Version 12.0 (ForteBio). Binding kinetics was evaluated using a 1:1 Langmuir model except for dimeric ACE2 and antibodies G32B6 and C12A2, which were analyzed by a bivalent binding model.

Flow cytometry

Expi293F cells (ThermoFisher Scientific) were grown in Expi293 expression medium (ThermoFisher Scientific). Cell surface display DNA constructs for the SARS-CoV-2 spike variants together with a plasmid expressing blue fluorescent protein (BFP) were transiently transfected into Expi293F cells using ExpiFectamine 293 reagent (ThermoFisher Scientific) per manufacturer's instruction. Two days after transfection, the cells were stained with primary antibodies or the histagged $ACE2_{615}$-foldon T27W protein (*42*) at 10 μg/ml concentration. For antibody staining, an Alexa Fluor 647 conjugated donkey anti-human IgG Fc F(ab')2 fragment (Jackson ImmunoResearch, West Grove, PA) was used as secondary antibody at 5 μg/ml concentration. For $ACE2_{615}$-foldon T27W staining, APC conjugated anti-HIS antibody (Miltenyi Biotec, Auburn, CA) was used as secondary antibody at 1:50 dilution. Cells were run through an

Intellicyt iQue Screener Plus flow cytometer. Cells gated for positive BFP expression were analyzed for antibody and $ACE2_{615}$-foldon T27W binding. The flow cytometry assays were repeated three times with essentially identical results.

HIV-based pseudovirus assay

Neutralizing activity against SARS-CoV-2 pseudovirus was measured using a single-round infection assay in 293T/ACE2 target cells. Pseudotyped virus particles were produced in 293T/17 cells (ATCC) by co-transfection of plasmids encoding codon-optimized SARS-CoV-2 full-length Spike constructs, packaging plasmid pCMV DR8.2, and luciferase reporter plasmid pHR' CMV-Luc. G614 Spike, packaging and luciferase plasmids were kindly provided by Dr. Barney Graham (Vaccine Research Center, NIH). The 293T cell line stably overexpressing the human ACE2 cell surface receptor protein was kindly provided by Drs. Michael Farzan and Huihui Ma (The Scripps Research Institute). For neutralization assays, serial dilutions of monoclonal antibodies (mAbs) were performed in duplicate followed by addition of pseudovirus. Pooled serum samples from convalescent COVID-19 patients or pre-pandemic normal healthy serum (NHS) were used as positive and negative controls, respectively. Plates were incubated for 1 hour at 37°C followed by addition of 293/ACE2 target cells (1x10^4/well). Wells containing cells + pseudovirus (without sample) or cells alone acted as positive and negative infection controls, respectively. Assays were harvested on day 3 using Promega BrightGlo luciferase reagent and luminescence detected with a Promega GloMax luminometer. Titers are reported as the concentration of mAb that inhibited 50% or 80% virus infection (IC_{50} and IC_{80} titers, respectively). All neutralization experiments were repeated twice with similar results.

Cryo-EM sample preparation and data collection

To prepare cryo grids, 3.5 µl of the freshly purified sample from the peak fraction in DDM at ~2.0 mg/ml for the B.1.1.7 protein or ~1.5 mg/ml for the B.1.351 protein was applied to a 1.2/1.3 Quantifoil grid (Quantifoil Micro Tools GmbH), which had been glow discharged with a PELCO easiGlow™ Glow Discharge Cleaning system (Ted Pella, Inc.) for 60 s at 15 mA. Grids were immediately plunge-frozen in liquid ethane using a Vitrobot Mark IV (ThermoFisher Scientific), and excess protein was blotted away by using grade 595 filter paper (Ted Pella, Inc.) with a blotting time of 4 s, a blotting force of -12 at 4°C in 100%

humidity. For data collection, images were acquired with selected grids using a Titan Krios transmission electron microscope (ThermoFisher Scientific) operated at 300 keV and equipped with a BioQuantum GIF/K3 direct electron detector. Automated data collection was carried out using SerialEM version 3.8.6 (*44*) at a nominal magnification of 105,000× and the K3 detector in counting mode (calibrated pixel size, 0.825 Å) at an exposure rate of 20.21 (for B.1.1.7) or 20.97 (for B.1.351) electrons per pixel per second. Each movie had a total accumulated electron exposure of ~53.40 (for B.1.1.7) or 55.46 (for B.1.351) e/Å^2, fractionated in 51 frames for both B.1.1.7 and B.1.351. Datasets were acquired using a defocus range of 1.4-2.3 µm (for B.1.1.7) or 1.0-2.3 µm (for B.1.351).

Image processing and 3D reconstructions

Drift correction for cryo-EM images was performed using MotionCor2 (*45*), and contrast transfer function (CTF) was estimated by CTFFIND4 (*46*) using motion-corrected sums without dose-weighting. Motion corrected sums with dose-weighting were used for all other image processing. RELION3.0.8 and crYOLO (*47*) were used for particle picking, 2D classification, 3D classification and refinement procedure. For the B.1.1.7 sample, approximately 3,000 particles were manually picked for each protein sample and subjected to 2D classification to generate the templates for automatic particle picking. After manual inspection of auto-picked particles, a total of 2,325,106 particles were extracted from 27,950 images. The selected particles were subjected to 2D classification, giving a total of 877,530 good particles. A low-resolution negative-stain reconstruction of the D614 S trimer (*22*) was low-pass filtered to 30Å resolution and used as an initial model for 3D classification with C1 symmetry. Three major classes showed clear structural features were subjected to another round of 3D classification with C1 symmetry, giving another three major classes. These three classes were joined together and re-extract to do one round of 3D auto-refinement, following by CTF Refinement, Particle Polishing, and another round of 3D auto-refinement, giving a map with 271,387 particles at 3.3Å resolution. Third round of signal-subtraction 3D classification without alignment at the apex region of the S trimer were performed to further classify, produced five different major classes, representing the closed, three RBD-down conformation, three of the one RBD-up conformations and a two RBD-up conformation, respectively. The five classes containing 13,919, 77,942, 7,368, 119,338, 41,138 particles,

respectively, were then subjected to another round of 3D auto-refinement with C1 (one RBD-up and two RBD-up) and C3 (closed) symmetry using an overall mask, resulting in five final reconstructions at 4.0Å, 3.3Å, 4.3Å, 3.2Å and 3.1Å resolutions, respectively. Several rounds of 3D auto-refinement was done for each then by adding different size of mask at the apex region to further improve the local resolution. The best map from each class was used for model building. A second independent data set gave essentially the same results except that there was no obvious class for the two RBD-up conformation.

For the B.1.351 sample, after manual inspection of auto-picked particles, a total of 2,869,130 particles were extracted from 22,448 images. The selected particles were subjected to three rounds of 2D classification, giving a total of 1,316,162 good particles. The low-resolution negative-stain reconstruction of the D614 S trimer was low-pass filtered to 30Å resolution and used as an initial model for 3D classification with C1 symmetry. After three rounds of 3D classification with C1 symmetry, one class represented the closed, three RBD-down conformation, and the rest of five classes all represented the one RBD-up conformations. The three RBD-down class was subjected to one round of 3D auto-refinement with C3 symmetry and particle polishing, followed by another round of 3D auto-refinement with C3 symmetry, giving a map at 3.1Å resolution. All five RBD-up classes were combined and subjected to one round of 3D auto-refinement with C1 symmetry and particle polishing, followed by another round of 3D auto-refinement with C1 symmetry, leading a map at 2.9Å resolution. A second independent data set also yielded very similar results.

The program cryoSPARC (*39*) was also used to independently validate 3D classification and refinement. Reported resolutions are based on the gold-standard Fourier shell correlation (FSC) using the 0.143 criterion. All density maps from RELION were corrected from the modulation transfer function of the K3 detector and then sharpened by applying a temperature factor that was estimated using post-processing in the program. Local resolution was determined using RELION with half-reconstructions as input maps.

Model building

The initial templates for model building used the stabilized SARS-CoV-2 S ectodomain trimer structure (PDB ID 7KRQ and PDB ID 7KRR) for both the B.1.351 and B.1.1.7 variant spike protein prefusion conformation. Several rounds of manual building were performed in Coot (*48*). The maps with a resolution lower than 4.0Å were primarily modeled manually in coot and by rigid body fitting, as the local resolution of many regions is higher than 4.0Å. The model was then refined in Phenix (*49*) against the 3.1Å (closed), 3.2Å, 3.3Å, 4.0Å (one RBD-up) and 4.3Å (two RBD-up) cryo-EM maps of the B.1.1.7 variant, and refined in Phenix against the 2.9Å (open), 3.1Å (closed) cryo-EM maps of the B.1.351 variant. Iteratively, refinement was performed in both Phenix (real space refinement) and ISOLDE (*50*), and the Phenix refinement strategy included minimization_global, local_grid_search, and adp, with rotamer, Ramachandran, and reference-model restraints, using 7KRQ and 7KRR as the reference model. The refinement statistics are summarized in Table S3. Structural biology applications used in this project were compiled and configured by SBGrid (*51*).

Supplementary Figures and Tables

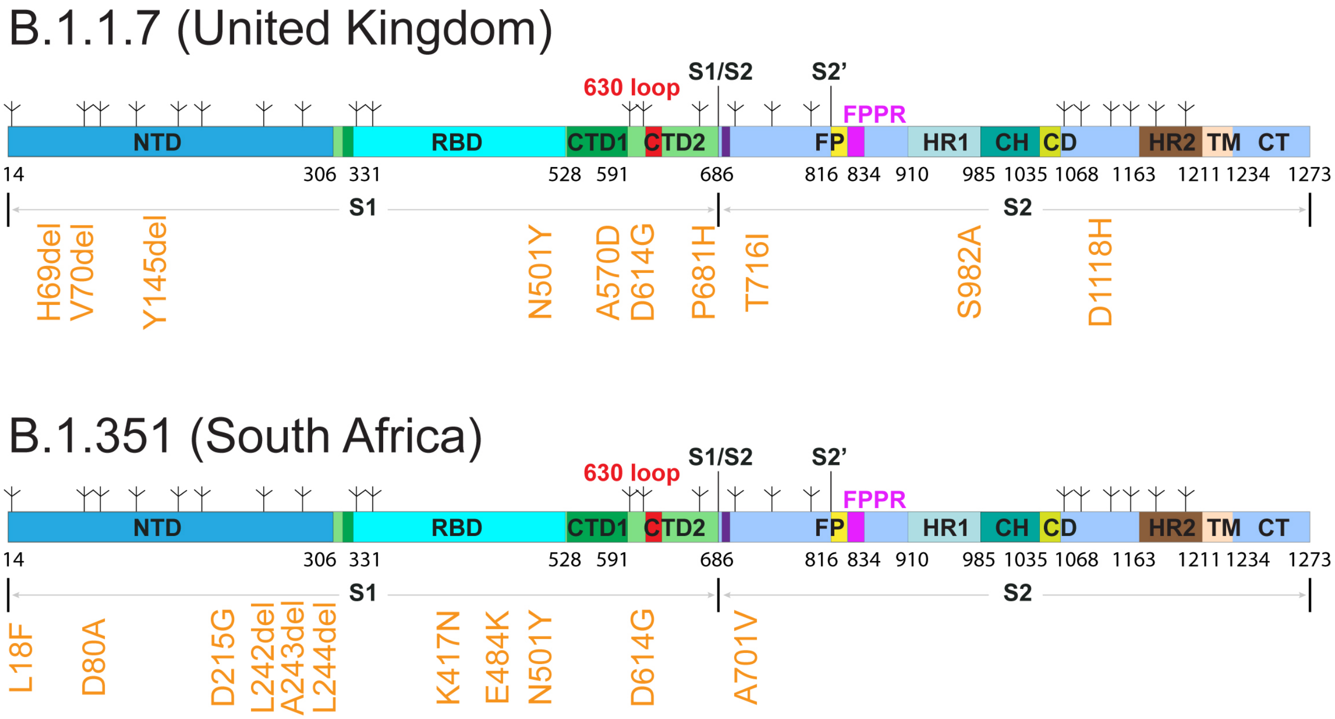

Figure S1. Schematic representation of the full-length SARS-CoV-2 spike (S) from the B.1.1.7 and B.1.351 variants. The sequences are derived from the B.1.1.7 (hCoV-19/England/MILK-C504CD/2020) and B.1.351 (hCoV-19/South Africa/KRISP-EC-MDSH925100/2020) variants. Segments of S1 and S2 include: NTD, N-terminal domain; RBD, receptor-binding domain; CTD1, C-terminal domain 1; CTD2, C-terminal domain 2; 630 loop; S1/S2, S1/S2 cleavage site; S2', S2' cleavage site; FP, fusion peptide; FPPR, fusion peptide proximal region; HR1, heptad repeat 1; CH, central helix region; CD, connector domain; HR2, heptad repeat 2; TM, transmembrane anchor; CT, cytoplasmic tail; and tree-like symbols for glycans. Positions of all mutations (from the amino-acid sequence of Wuhan-Hu-1) are shown in orange text.

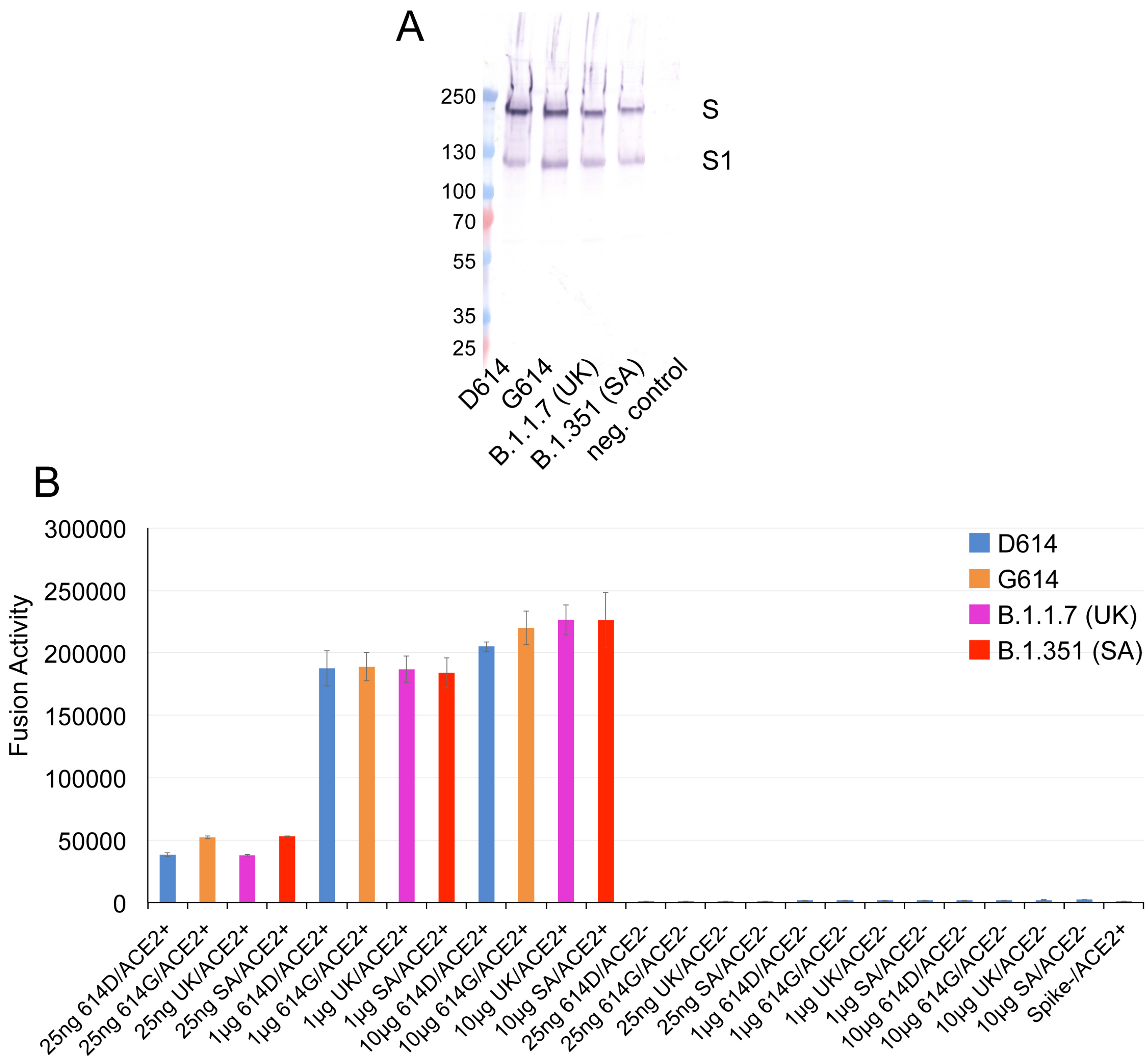

Figure S2. Expression and cell-cell fusion of SARS-CoV-2 variants. (**A**) Expression and processing of the full-length S constructs in HEK293 cells. S samples prepared from HEK293 cells transiently transfected with 10 μg of the full-length S expression plasmids were detected by anti-RBD polyclonal antibodies. Bands for the uncleaved S and S1 fragment are indicated. (**B**) HEK293T cells transfected with either the untagged full length S protein expression plasmids were fused with ACE2-expressing cells. Cell-cell fusion led to reconstitution of α and ω

fragments of β-galactosidase yielding an active enzyme, and the fusion activity was then quantified by a chemiluminescent assay. No ACE2 and no S were negative controls.

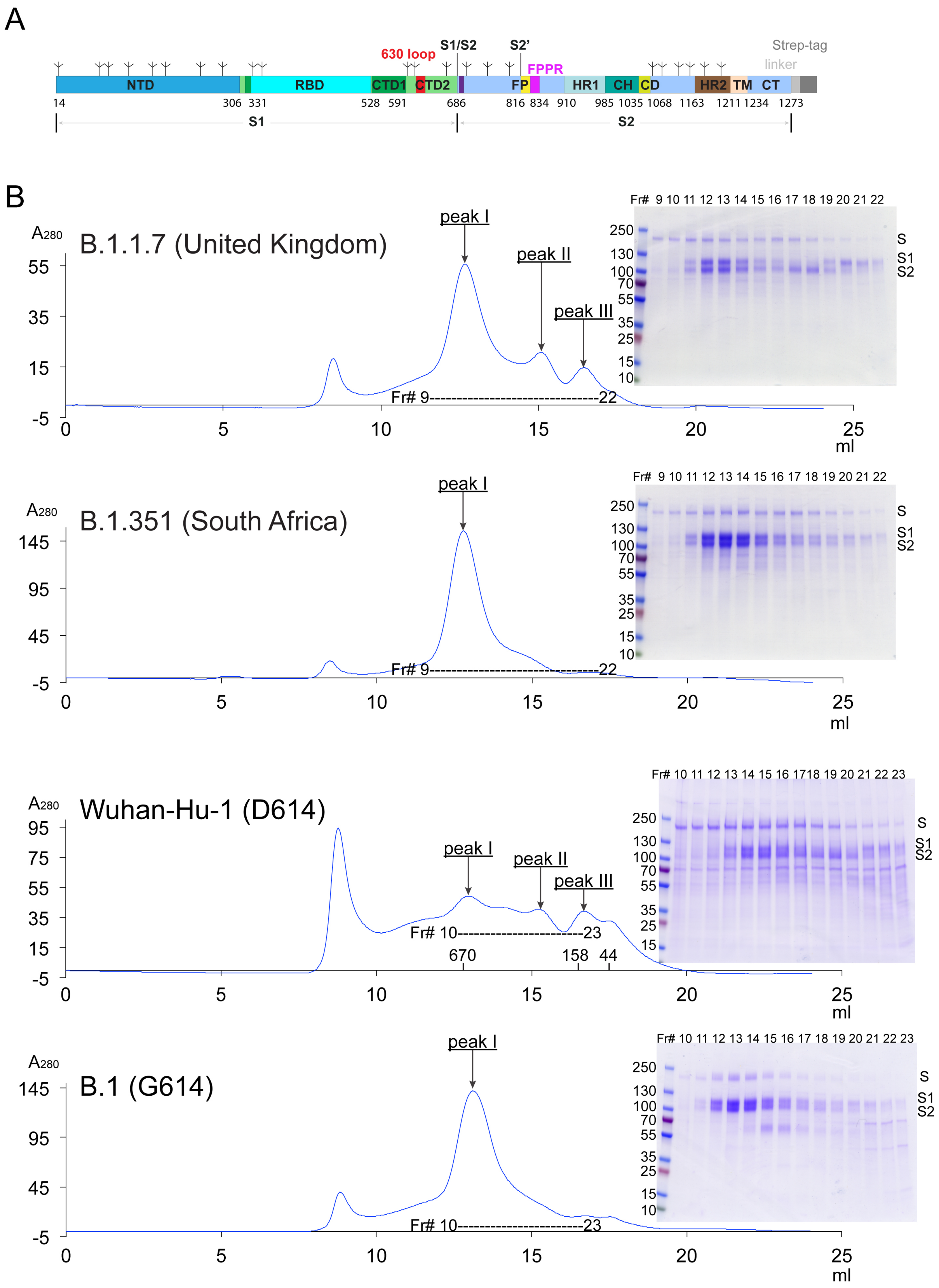

A
630 loop
S1/S2
S2'
Strep-tag
linker
FPPR
NTD
RBD
CTD1
CTD2
FP
HR1
CH
CD
HR2
TM
CT
14 306 331 528 591 686 816 834 910 985 1035 1068 1163 1211 1234 1273
S1
S2
B
A280
B.1.1.7 (United Kingdom)
peak I
peak II
peak III
Fr# 9-----22
ml
Fr# 9 10 11 12 13 14 15 16 17 18 19 20 21 22
250 130 100 70 55 35 25 15 10
S
S1
S2
B.1.351 (South Africa)
Wuhan-Hu-1 (D614)
Fr# 10-----23
670
158
44
Fr# 10 11 12 13 14 15 16 17 18 19 20 21 22 23
B.1 (G614)

Figure S3. Production of full-length S protein from the B.1.1.7 and B.1.351 variants. (**A**) A strep-tag was fused to the C-terminus of the full-length S protein by a flexible linker. (**B**) The full-length S proteins were extracted and purified in detergent DDM, and further resolved by gel-filtration chromatography on a Superose 6 column. Peak I, the prefusion S trimer; peak II, the postfusion S2 trimer; and peak III, the dissociated monomeric S1. Inset, peak fractions were analyzed by Coomassie stained SDS-PAGE. Labeled bands are S, S1 and S2. Fr#, fraction number. Each experiment was repeated at least three times independently with similar results. The data for the preparations from the Wuhan-Hu-1 (D614) and B.1 (G614) are included for convenient comparison and were previously published (*22, 28*).

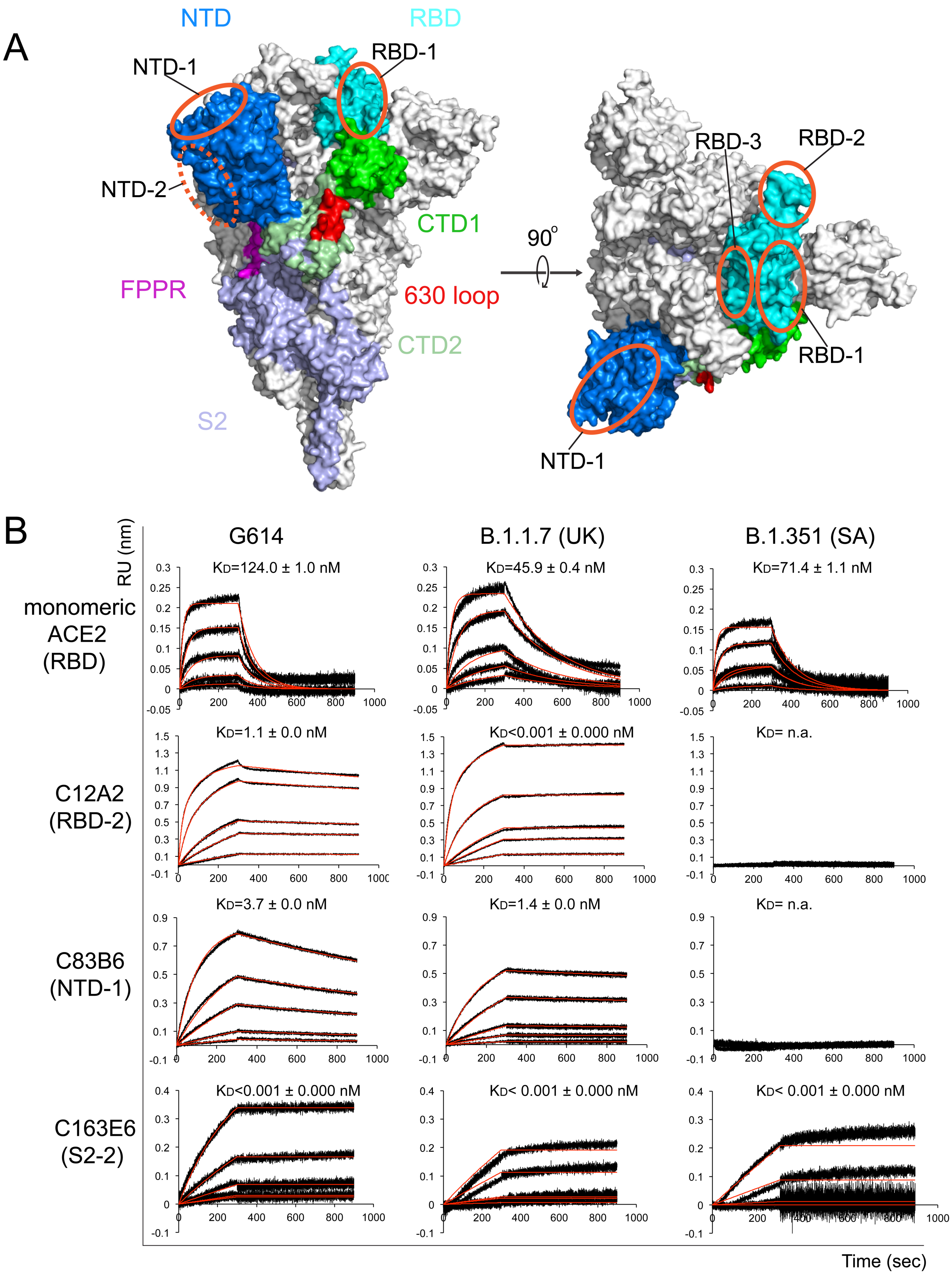
A
NTD
RBD
NTD-1
RBD-1
NTD-2
CTD1
FPPR
630 loop
CTD2
S2
90°
RBD-3
RBD-2
RBD-1
NTD-1
B
G614
B.1.1.7 (UK)
B.1.351 (SA)
RU (nm)
monomeric ACE2 (RBD)
KD=124.0 ± 1.0 nM
KD=45.9 ± 0.4 nM
KD=71.4 ± 1.1 nM
C12A2 (RBD-2)
KD=1.1 ± 0.0 nM
KD<0.001 ± 0.000 nM
KD= n.a.
C83B6 (NTD-1)
KD=3.7 ± 0.0 nM
KD=1.4 ± 0.0 nM
KD= n.a.
C163E6 (S2-2)
KD<0.001 ± 0.000 nM
KD< 0.001 ± 0.000 nM
KD< 0.001 ± 0.000 nM
Time (sec)

Figure S4. Additional antigenic properties of the purified full-length SARS-CoV-2 S proteins. (**A**) Antibody competition clusters as described in ref(*35*). Surface regions of the S trimer targeted by antibodies on S1 are highlighted by orange ellipses, including RBD-1, RBD-2, RBD-3, NTD-1 and NTD-2. The exact location of NTD-2 is uncertain and therefore marked with a dashed line. (**B**) Binding analysis of the prefusion S trimers from G614, B.1.1.7 and B.1.351 variants with soluble ACE2 constructs was performed by bio-layer interferometry (BLI). For ACE2 binding, the purified S proteins were immobilized on AR2G biosensors and dipped into the wells containing ACE2 at various concentrations. For antibody binding, various antibodies were immobilized to AHC biosensors and dipped into the wells containing each purified S protein at different concentration. Binding kinetics were evaluated using a 1:1 Langmuir model except for antibody C12A2 targeting the RBD-2, which was analyzed by a bivalent binding model. The sensorgrams are in black and the fits in red. RU, response unit. Binding constants are also summarized here and in Table S1. All experiments were repeated at least twice with essentially identical results.

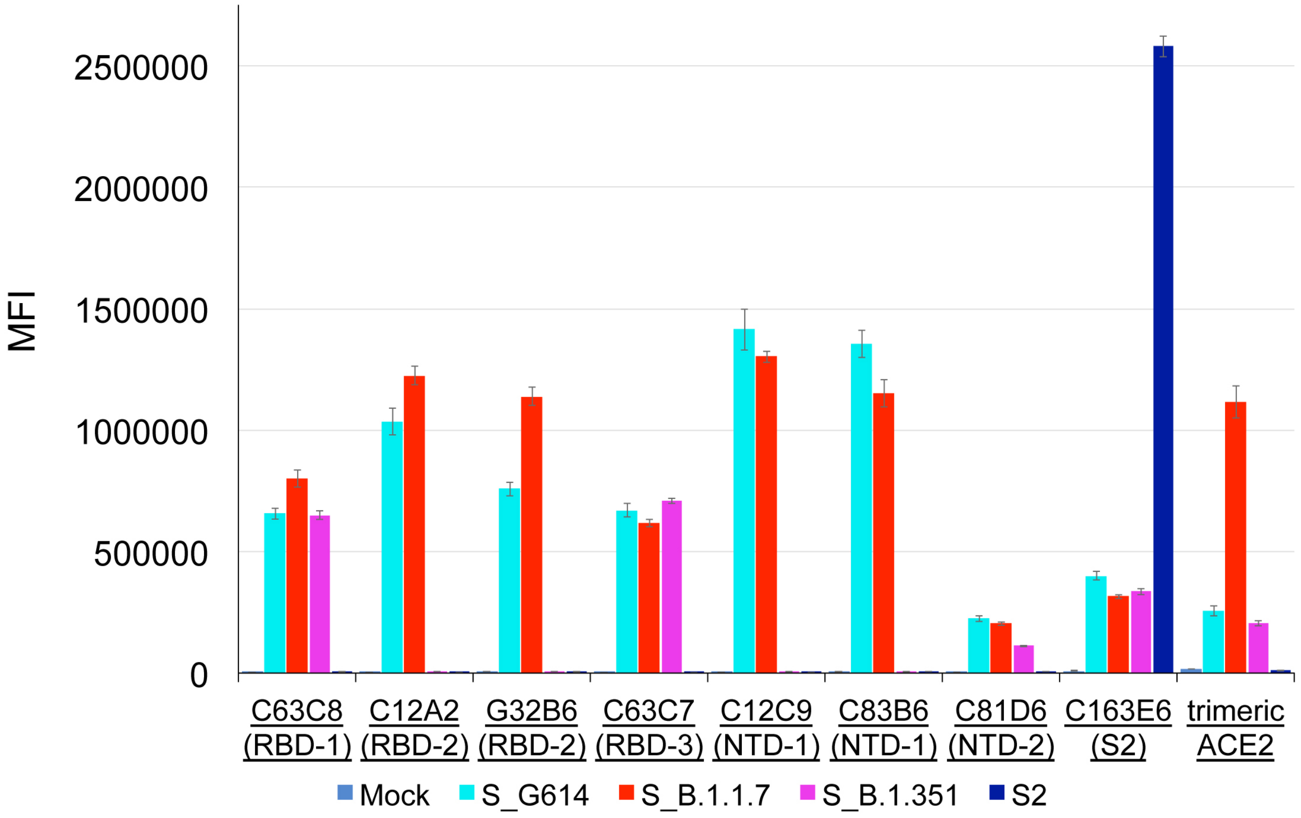

Figure S5. Antigenic properties of the cell-surface S proteins assessed by flow cytometry. Antibody and ACE2 binding to the full-length S proteins of the G614, B.1.1.7 and B.1.351 variants, as well as an S2 construct expressed on the cell surfaces analyzed by flow cytometry. The antibodies and their targets are indicated. A designed ACE2-based inhibitor $ACE2_{615}$-foldon-T27W was used for detecting receptor binding (*42*). MFI, mean fluorescent intensity. The error bars represent standard errors of mean from measurements using three independently transfected cell samples. The flow cytometry assays were repeated three times with essentially identical results.

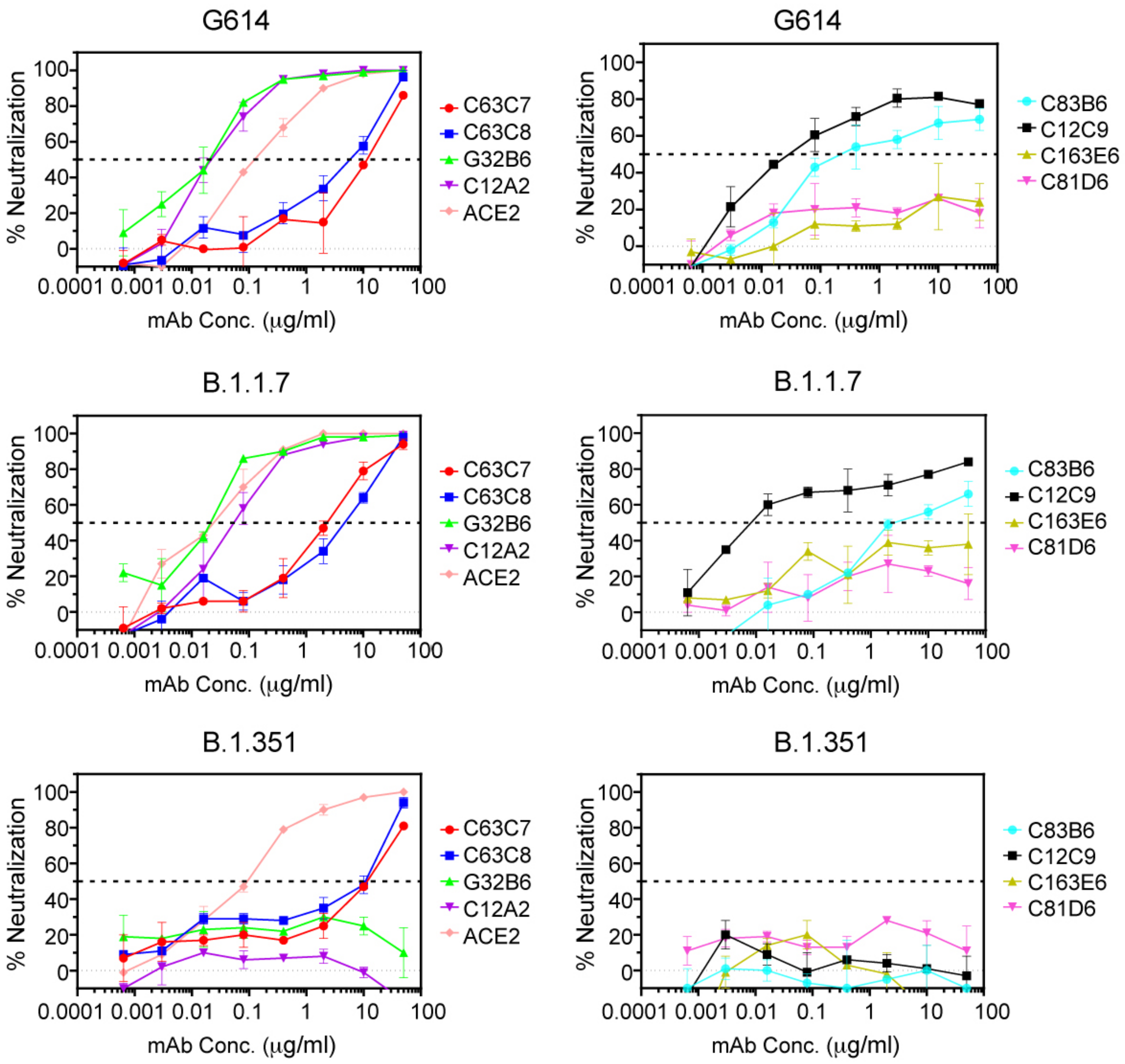

Figure S6. Neutralization of the SARS-CoV-2 variants by a pseudovirus assay. Serial dilutions of various monoclonal antibodies and a designed trimeric ACE2 construct, ACE2-T27W-Fd, were tested for neutralization against HIV-based pseudotyped viruses expressing full-length SARS-CoV-2 G614 S, B.1.1.7 S or B.1.351 S. IC_{50} values were derived from curve fitting and summarized in Table S2. The experiments were repeated three times with independent samples giving similar results.

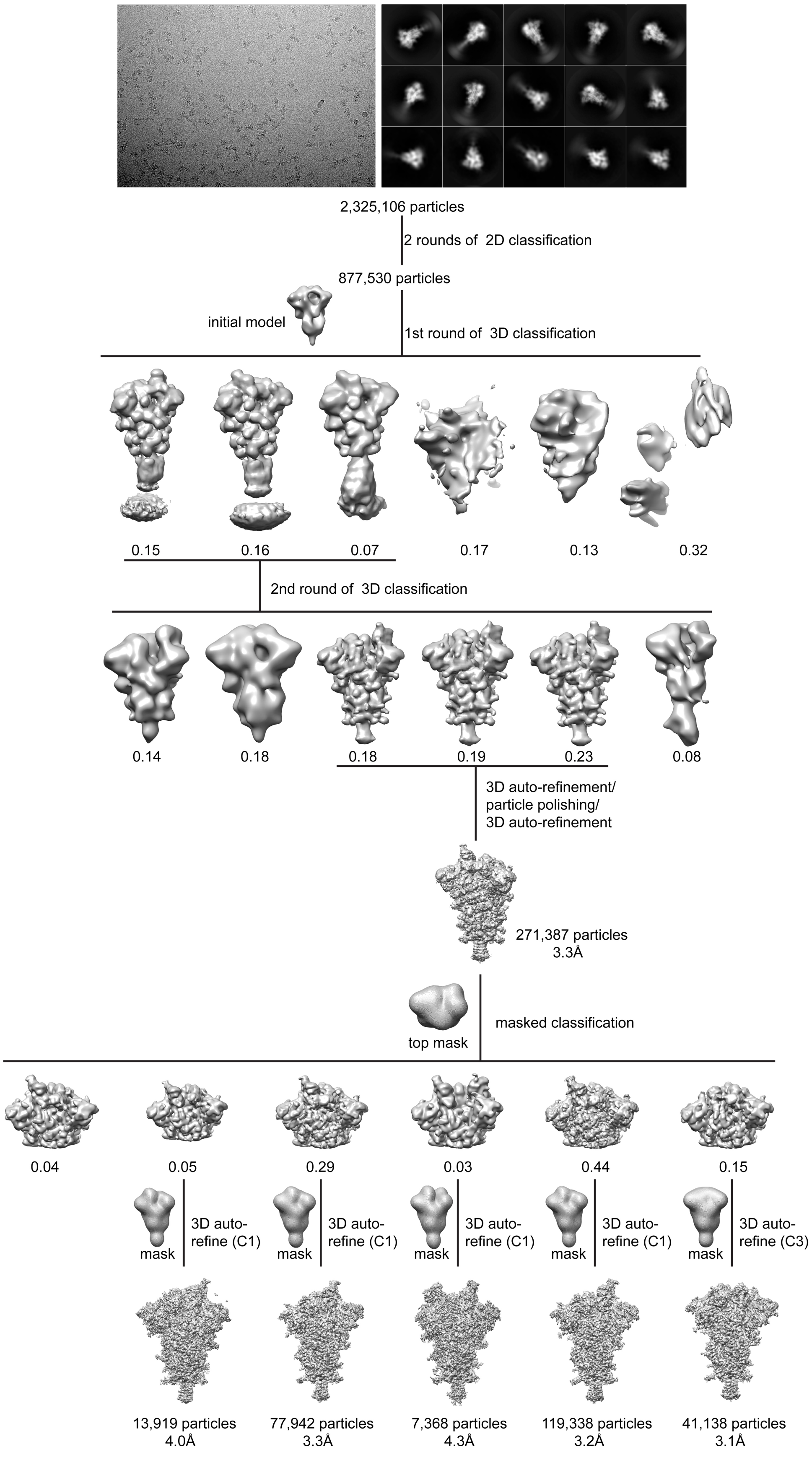
2,325,106 particles
2 rounds of 2D classification
877,530 particles
initial model
1st round of 3D classification
0.15
0.16
0.07
0.17
0.13
0.32
2nd round of 3D classification
0.14
0.18
0.18
0.19
0.23
0.08
3D auto-refinement/
particle polishing/
3D auto-refinement
271,387 particles
3.3Å
top mask
masked classification
0.04
0.05
0.29
0.03
0.44
0.15
mask
3D auto-refine (C1)
mask
3D auto-refine (C1)
mask
3D auto-refine (C1)
mask
3D auto-refine (C1)
mask
3D auto-refine (C3)
13,919 particles
4.0Å
77,942 particles
3.3Å
7,368 particles
4.3Å
119,338 particles
3.2Å
41,138 particles
3.1Å

Figure S7. Cryo-EM analysis of the B.1.1.7 S trimer. Top, representative micrograph, and 2D averages (box dimension: 396Å) of the cryo-EM particle images of the B.1.1.7 S trimer. Bottom, data processing workflow for structure determination.

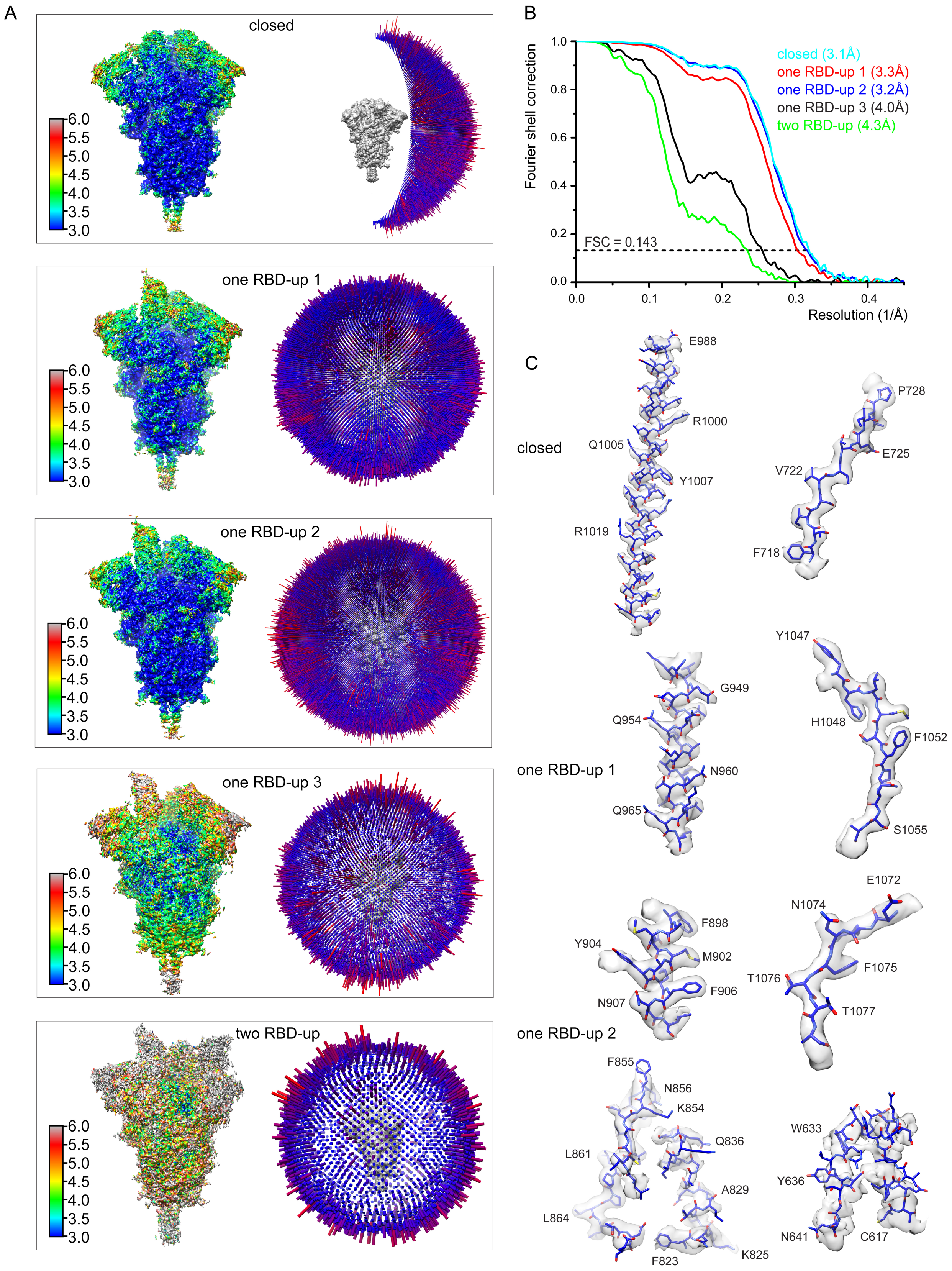

A
closed
one RBD-up 1
one RBD-up 2
one RBD-up 3
two RBD-up
6.0
5.5
5.0
4.5
4.0
3.5
3.0
B
Fourier shell correction
closed (3.1Å)
one RBD-up 1 (3.3Å)
one RBD-up 2 (3.2Å)
one RBD-up 3 (4.0Å)
two RBD-up (4.3Å)
FSC = 0.143
Resolution (1/Å)
C
closed
E988
R1000
Q1005
Y1007
R1019
P728
E725
V722
F718
one RBD-up 1
G949
Q954
N960
Q965
Y1047
H1048
F1052
S1055
one RBD-up 2
F898
Y904
M902
F906
N907
E1072
N1074
F1075
T1076
T1077
F855
N856
K854
Q836
L861
A829
L864
F823
K825
W633
Y636
N641
C617

Figure S8. Analysis of the B.1.1.7 S trimer structure. (**A**) 3D reconstructions of the B.1.1.7 S trimer preparation in the closed, three one RBD-up and a two RBD-up conformations, respectively, are colored according to local resolution estimated by RELION. Angular distribution of the cryo-EM particles used in each reconstruction is shown in the side view of the EM map. (**B**) Gold standard FSC curves of the three refined 3D reconstructions of the B.1.1.7 S trimer. (**C**) Representative density in gray surface from EM maps with a resolution better than 3.5Å.

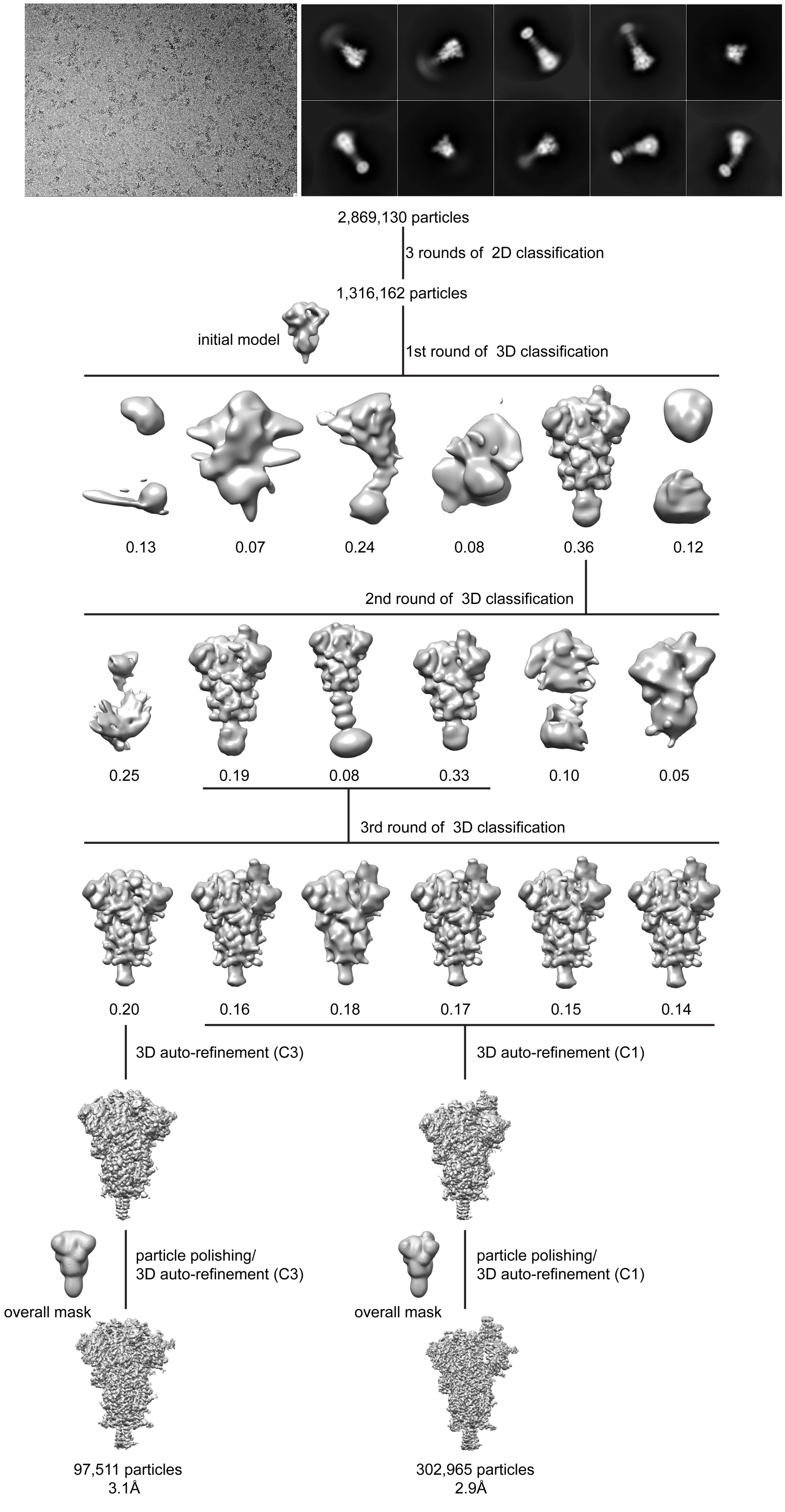

2,869,130 particles
3 rounds of 2D classification
1,316,162 particles
initial model
1st round of 3D classification
0.13
0.07
0.24
0.08
0.36
0.12
2nd round of 3D classification
0.25
0.19
0.08
0.33
0.10
0.05
3rd round of 3D classification
0.20
0.16
0.18
0.17
0.15
0.14
3D auto-refinement (C3)
3D auto-refinement (C1)
particle polishing/
3D auto-refinement (C3)
overall mask
particle polishing/
3D auto-refinement (C1)
overall mask
97,511 particles
3.1Å
302,965 particles
2.9Å

Figure S9. Cryo-EM analysis of the B.1.351 S trimer. Top, representative micrograph, and 2D averages (box dimension: 396Å) of the cryo-EM particle images of the B.1.351 S trimer. Bottom, data processing workflow for structure determination.

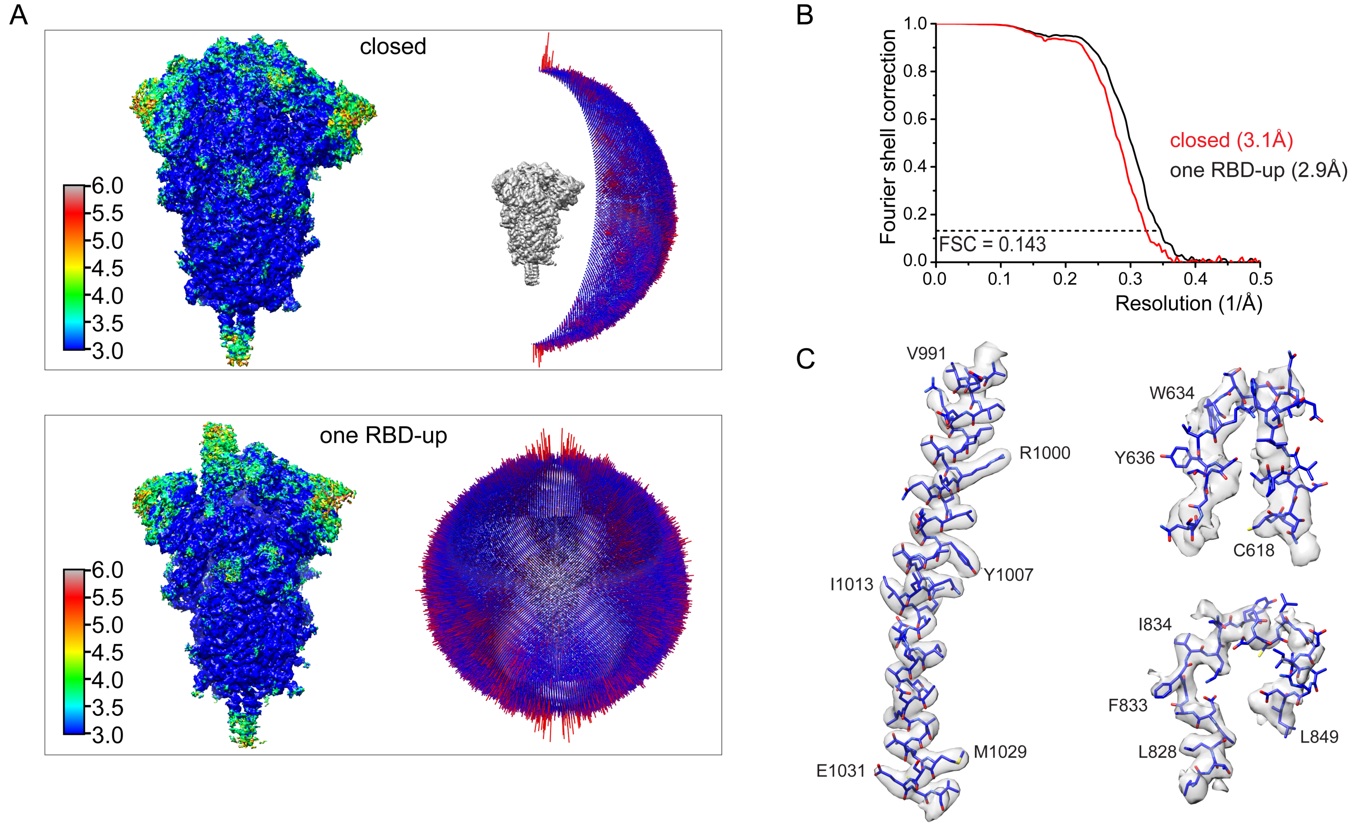

Figure S10. Analysis of the B.1.351 S trimer structure. (**A**) 3D reconstructions of the B.1.351 S trimer preparation in the closed and one RBD-up conformations, respectively, are colored according to local resolution estimated by RELION. Angular distribution of the cryo-EM particles used in reconstruction for the closed conformation is shown in the side view of the EM map. (**B**) Gold standard FSC curves of the refined 3D reconstructions of the B.1.351 S trimer. (**C**) Representative density in gray surface from EM map of the one RBD-up structure.

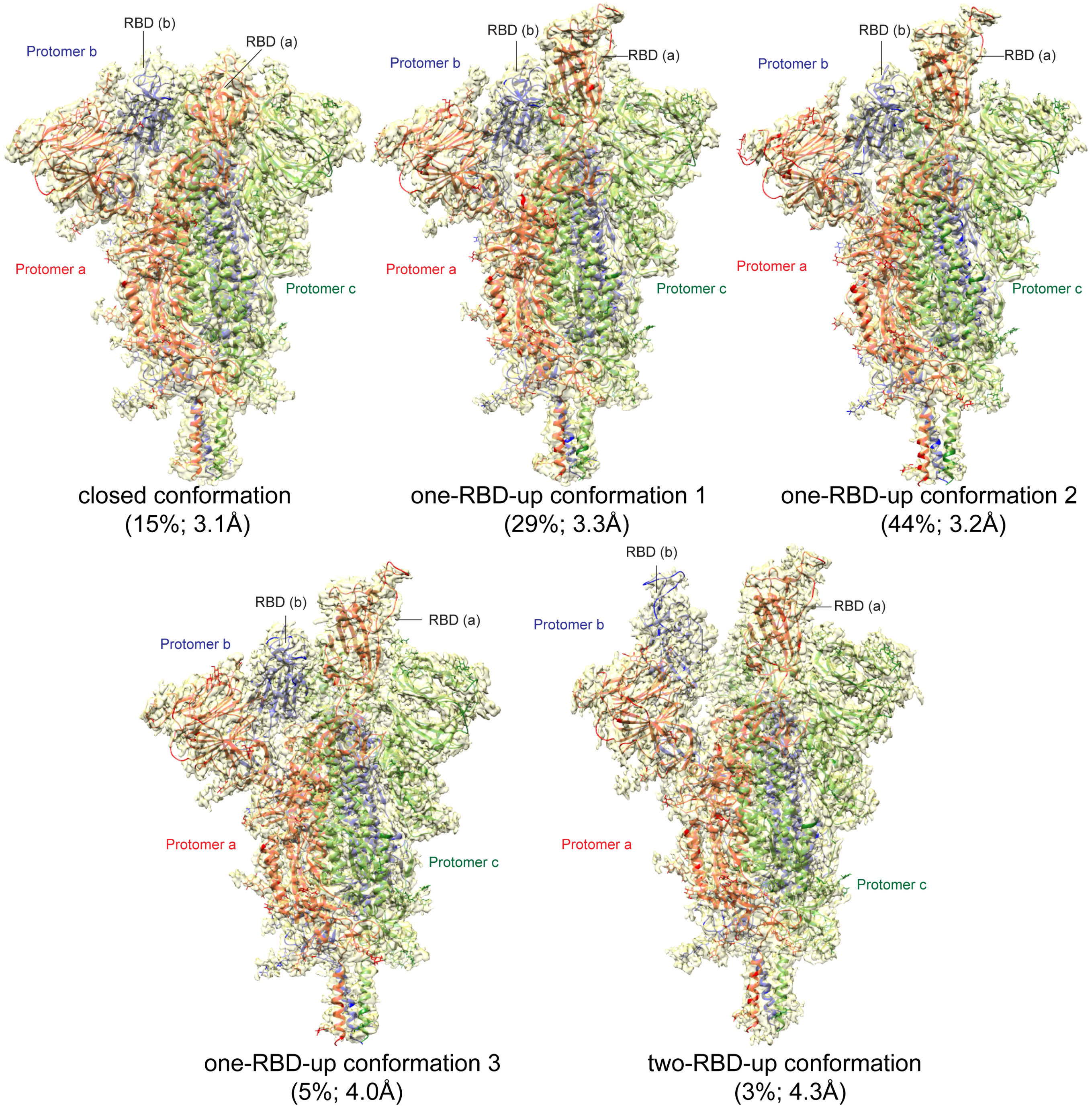

Figure S11. Cryo-EM structures of the full-length S protein of the B.1.1.7 variant. Five structures of the B.1.1.7 S trimer, representing a closed prefusion conformation, three distinct RBD-up conformations, and a two RBD-up conformation, were modeled based on corresponding cryo-EM density maps at 3.1-4.3Å resolution. The maps with a resolution lower than 4.0Å were primarily modeled manually in coot and by rigid body fitting, as the local resolution of many

regions is higher than 4.0Å. Three protomers (a, b, c) are colored in red, blue and green, respectively. RBD locations are indicated. Particle percentage for each class in the data processing is also indicated, but it may not accurately reflect the conformation distribution of the S trimer in solution.

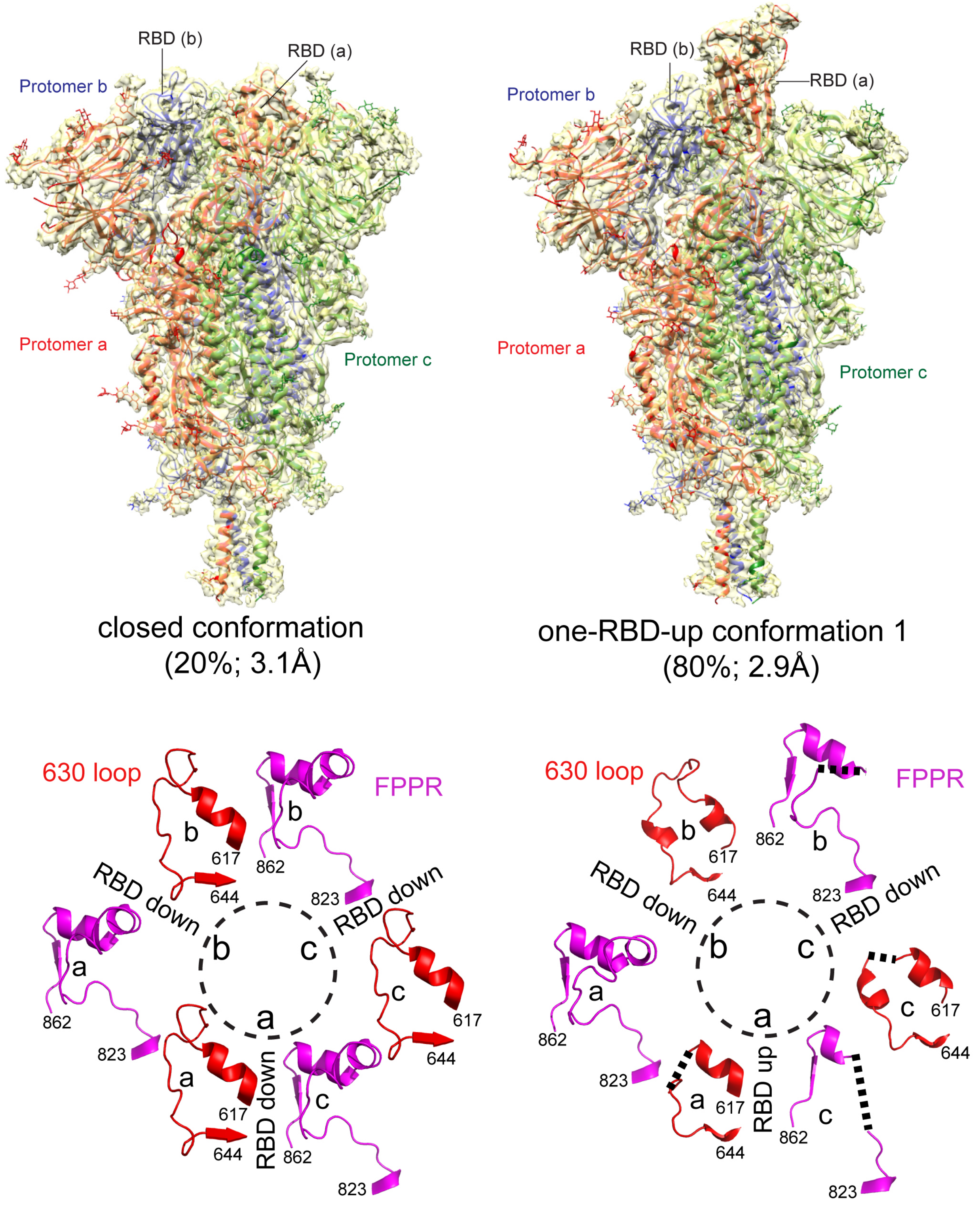

Figure S12. Cryo-EM structures of the full-length S protein of the B.1.351 variant. Top, two structures of the B.1.351 S trimer, representing a closed prefusion conformation, and an RBD-up

conformation were modeled based on corresponding cryo-EM density maps at 3.1Å and 2.9Å resolution, respectively. Three protomers (a, b, c) are colored in red, blue and green, respectively. RBD locations are indicated. Particle percentage for each class in the data processing is also indicated, but it may not accurately reflect the conformation distribution of the S trimer in solution. Bottom, structures of three segments (residues 617-644) containing the 630 loop in red and three segments (residues 823-862) containing the FPPR in magenta from all three protomers (a, b and c) are shown for the B.1.1.7 trimer. Position of each RBD is indicated. Dashed lines indicate gaps.

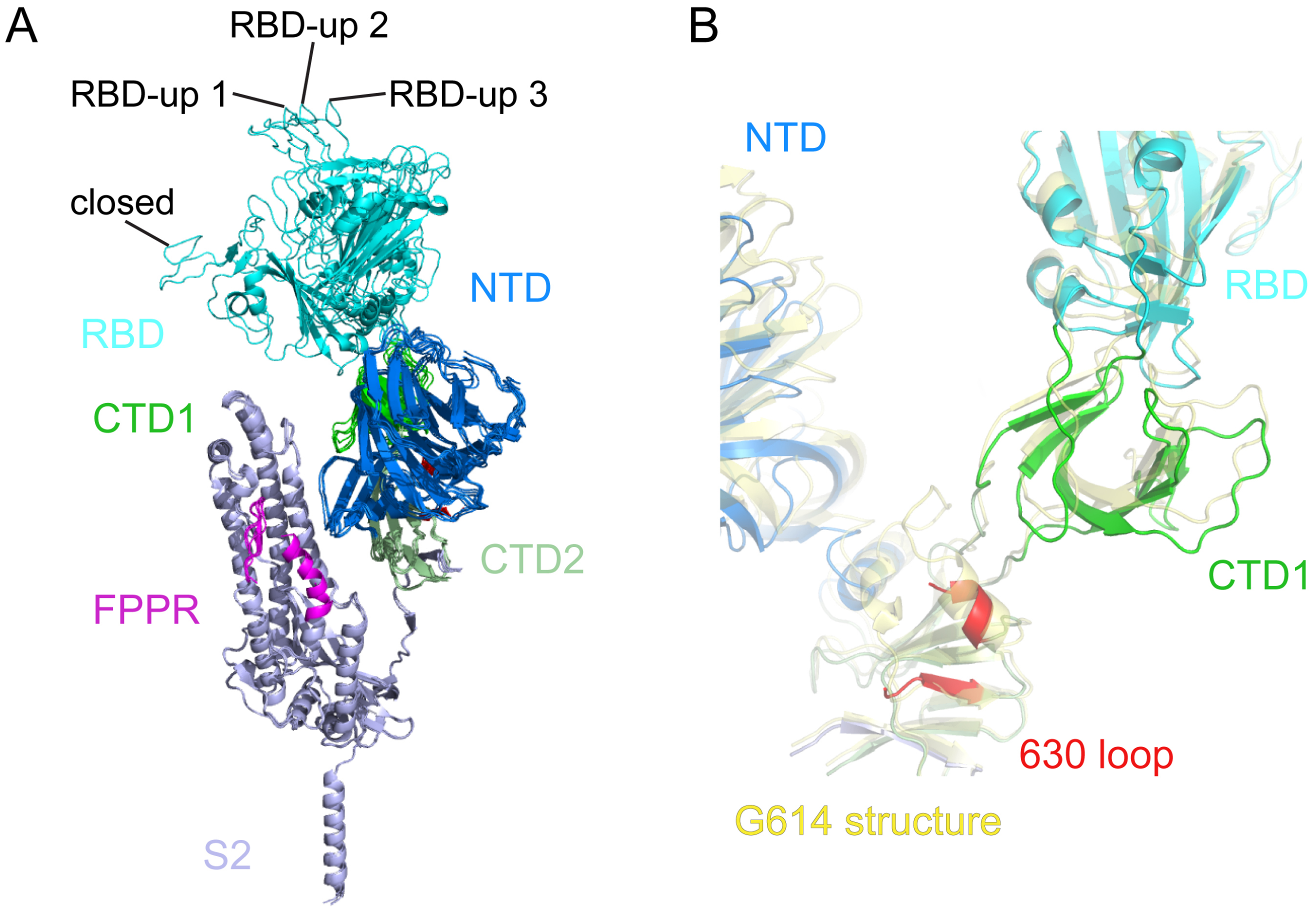

Figure S13. Superposition of the B.1.1.7 trimer structures and the G614 structure. (**A**) Side views of superposition of the closed conformation and three distinct one RBD-up conformations of the B.1.1.7 S in ribbon, align by the S2 portion. The positions of the RBD-down and three different RBD-up conformations are indicated. (**B**) Comparison of the CTD1 in the B.1.1.7 (various colors) and G614 trimer (yellow) structures, when aligned by S2.

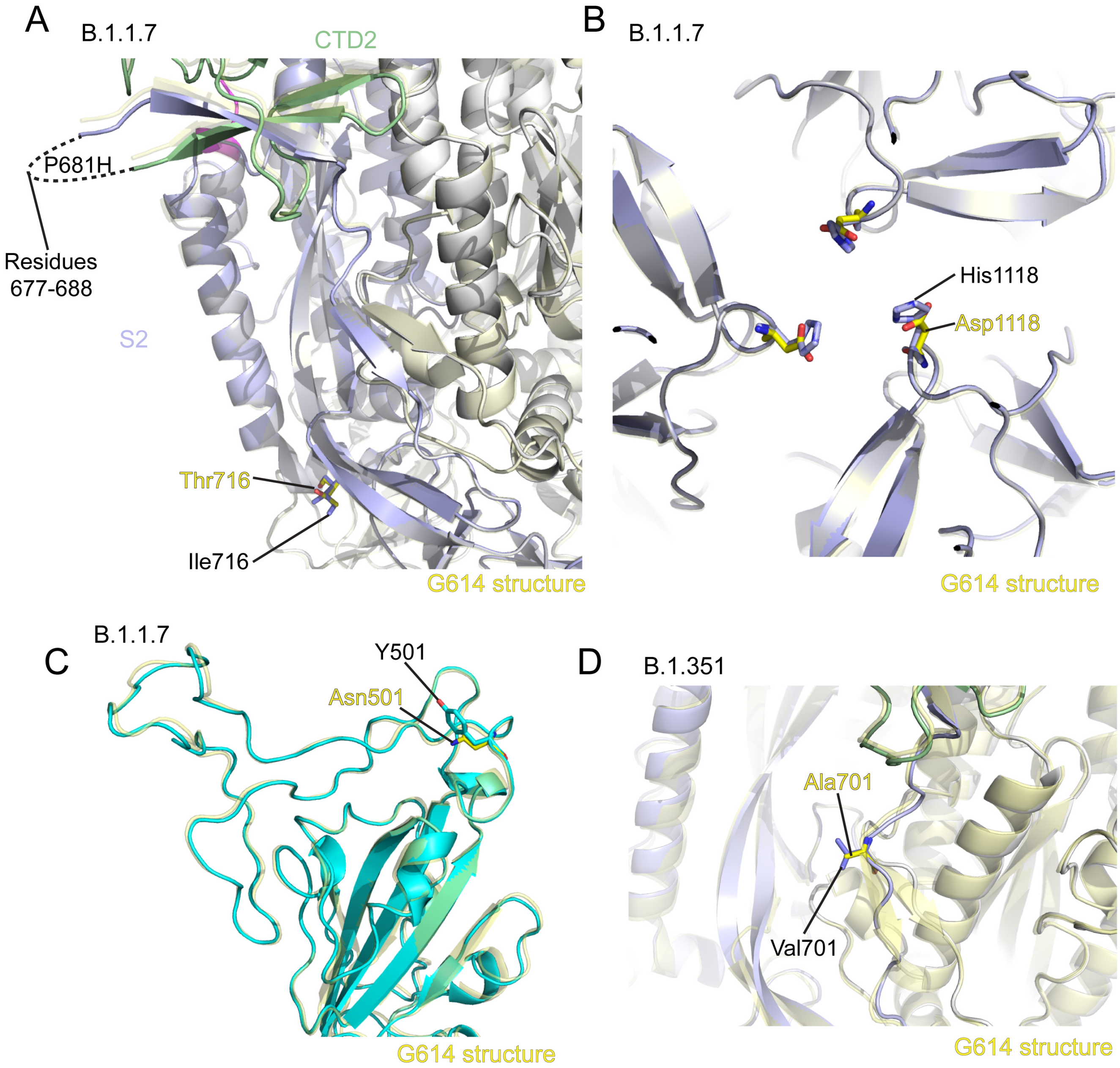

Figure S14. Structural impact of the mutations in the variants. (**A-C**) Views of superposition of the structure of the B.1.1.7 S trimer in ribbon representation with the structure of the G614 S in yellow, showing the regions near mutations N501Y, P681H, T716I and D1118H. (**D**) A view of superposition of the structure of the B.1.351 S trimer in ribbon representation with the structure of the G614 S in yellow, showing the region near the mutation A701V. All mutations are indicated and highlighted as sticks.

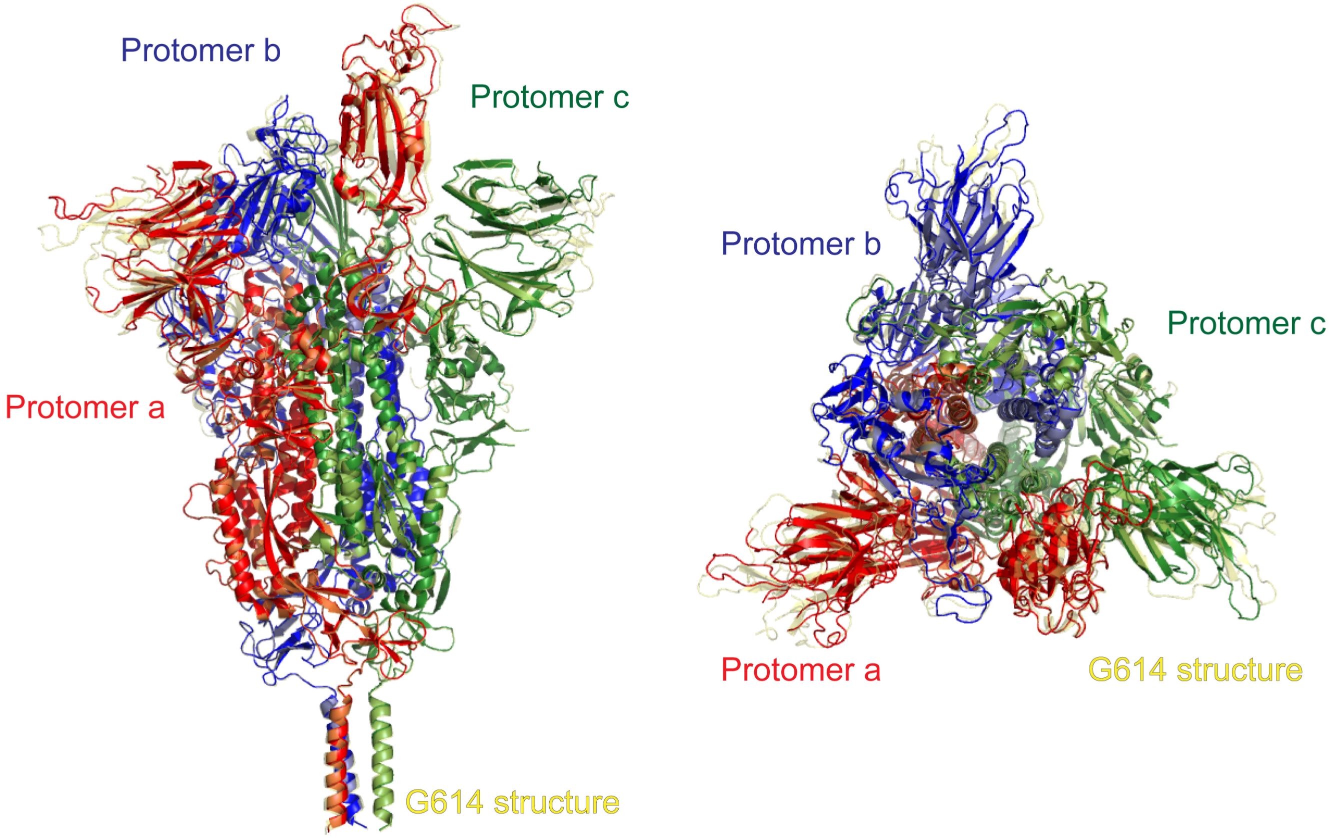

Figure S15. Superposition of the structures of the B.1.351 and G614 S trimers in the one RBD-up conformation. The structures of the B.1.351 and G614 S (PDB ID: 7KRR) trimers in the one RBD-up conformation are aligned by the invariant S2. Three protomers (a, b, c) are colored in red, blue and green, respectively.

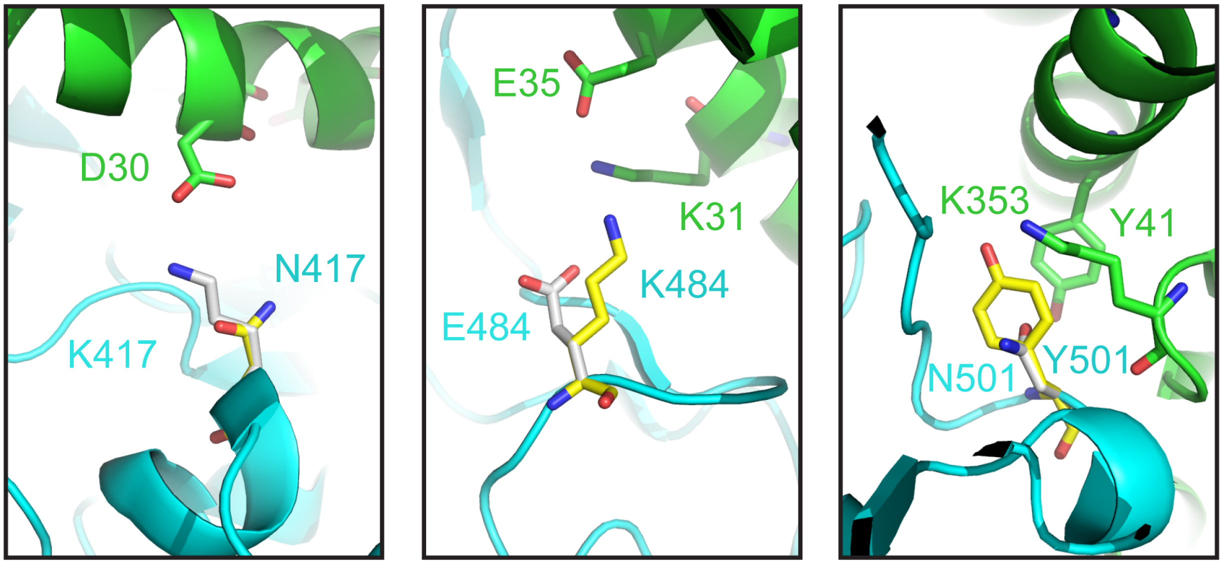

Figure S16. Modeled interface between the RBD and ACE2. The interface between ACE2 in ribbon diagram in green and RBD in cyan from the complex structure (PDB ID: 6M0J; ref(*17*)). Modeled K417N, E484K and N501Y are shown as sticks.

Table S1. Binding constants of S-ACE2 interaction

		K_D (M)	K_D Error	ka (1/Ms)	ka2	ka Error	ka2 Error	kdis (1/s)	kdis2	kdis Error	kdis2 Error
CE2-Fc	G614	1.28E-08	6.33E-10	4.30E+04	6.39E+00	1.05E+03	2.03E+00	5.48E-04	9.58E-01	2.37E-05	2.88E-01
RBD)	B.1.1.7(UK)	2.67E-09	2.03E-10	7.26E+04	3.51E-01	3.50E+03	3.15E-01	1.94E-04	2.93E-02	1.14E-05	2.76E-02
	B.1.351(SA)	1.51E-08	8.40E-10	4.96E+04	1.08E+01	1.09E+03	1.52E+02	7.47E-04	3.99E-01	3.83E-05	5.56E+00
nomeric	G614	1.24E-07	1.04E-09	9.65E+04		6.81E+02		1.20E-02		5.43E-05	
CE2	B.1.1.7(UK)	4.59E-08	3.99E-10	7.61E+04		6.34E+02		3.49E-03		8.84E-06	
RBD)	B.1.351(SA)	7.14E-08	1.11E-09	1.15E+05		1.72E+03		8.22E-03		3.58E-05	
63C8	G614	1.55E-08	8.07E-11	6.07E+04		2.93E+02		9.41E-04		1.81E-06	
BD-1)	B.1.1.7(UK)	2.59E-09	1.29E-11	1.09E+05		2.84E+02		2.81E-04		1.19E-06	
	B.1.351(SA)	2.61E-08	3.33E-10	3.72E+04		4.59E+02		9.70E-04		3.13E-06	
32B6	G614	1.40E-09	1.32E-11	1.98E+05	2.50E-01	1.06E+03	3.02E-02	2.78E-04	4.50E-02	2.17E-06	5.28E-03
BD-2)	B.1.1.7(UK)	2.20E-10	2.02E-12	1.04E+05	2.15E+00	4.46E+02	2.02E-02	2.28E-05	5.97E-02	1.85E-07	4.33E-04
	B.1.351(SA)	N.D.	N.D.	N.D.	N.D.	N.D.	N.D.	N.D.	N.D.	N.D.	N.D.
12A2	G614	1.13E-09	1.66E-11	2.02E+05	3.64E-01	1.72E+03	8.82E-02	2.28E-04	5.67E-02	2.72E-06	1.32E-02
BD-2)	B.1.1.7(UK)	<1.0E-12	4.12E-11	9.05E+04	3.23E-01	7.83E+02	2.04E-01	<1.0E-07	1.01E-02	3.73E-06	5.18E-03
	B.1.351(SA)	N.D.	N.D.	N.D.	N.D.	N.D.	N.D.	N.D.	N.D.	N.D.	N.D.
63C7	G614	1.09E-08	7.70E-11	8.81E+04		5.56E+02		9.64E-04		2.99E-06	
BD-3)	B.1.1.7(UK)	3.44E-09	2.07E-11	9.60E+04		3.60E+02		3.30E-04		1.55E-06	
	B.1.351(SA)	1.18E-08	7.07E-11	1.32E+05		7.33E+02		1.56E-03		3.55E-06	
12C9	G614	1.10E-08	5.46E-11	4.68E+04		2.05E+02		5.13E-04		1.22E-06	
TD-1)	B.1.1.7(UK)	5.84E-10	4.43E-12	2.16E+05		4.15E+02		1.26E-04		9.26E-07	
	B.1.351(SA)	N.D.		N.D.		N.D.		N.D.		N.D.	
83B6	G614	3.72E-09	1.11E-11	1.26E+05		2.58E+02		4.70E-04		1.02E-06	
TD-1)	B.1.1.7(UK)	1.38E-09	1.75E-11	8.03E+04		2.96E+02		1.11E-04		1.35E-06	
	B.1.351(SA)	N.D.		N.D.		N.D.		N.D.		N.D.	
81D6	G614	3.20E-09	1.99E-11	1.28E+05		5.05E+02		4.08E-04		1.96E-06	
TD-2)	B.1.1.7(UK)	3.15E-09	4.54E-11	4.35E+04		3.20E+02		1.37E-04		1.69E-06	
	B.1.351(SA)	4.87E-09	4.92E-11	4.74E+04		3.18E+02		2.31E-04		1.75E-06	
163E6	G614	<1.0E-12	2.78E-12	5.50E+04		3.95E+02		<1.0E-07		1.53E-07	
S2-2)	B.1.1.7(UK)	<1.0E-12	4.24E-11	9.32E+03		9.56E+02		<1.0E-07		3.95E-07	
	B.1.351(SA)	<1.0E-12	1.17E-09	6.02E+03		1.19E+03		<1.0E-07		7.07E-06	

Table S2. Neutralization of the SARS-CoV-2 variants

**Antibody/ACE2 construct**	**Neutralization titer (μg/ml)**					
	**G614**		**B.1.1.7 (UK)**		**B.1.351 (South Africa)**	
	IC_{50}	IC_{80}	IC_{50}	IC_{80}	IC_{50}	IC_{80}
C63C8 (RBD-1)	3.815	31.072	3.148	37.899	7.570	31.210
C12A2 (RBD-2)	0.020	0.080	0.047	0.204	>50	>50
G32B6 (RBD-2)	0.019	0.074	0.022	0.069	>50	>50
C63C7 (RBD-3)	8.081	44.520	2.067	17.671	11.450	48.346
C12C9 (NTD-1)	0.023	4.045	0.015	4.278	>50	>50
C83B6 (NTD-1)	0.189	>50	4.541	>50	>50	>50
C81D6 (NTD-2)	>50	>50	>50	>50	>50	>50
C163E6 (S2-2)	>50	>50	>50	>50	>50	>50
ACE2-T27W-Fd	0.138	0.774	0.028	0.157	0.082	0.557
Positive serum pool_2 (1/x)	753	132	711	103	41	<20
Normal Human Serum (1/x)	<20	<20	<20	<20	<20	<20

Table S3. Cryo-EM statistics.

EM data collection and reconstruction statistics

Protein	Full-length B.1.351 S protein		Full-length B.1.1.7 S protein				
Detergent	DDM		DDM				
Microscope	Titan Krios		Titan Krios				
Voltage(kV)	300		300				
Detector	Gatan K3		Gatan K3				
Magnification(nominal)	105,000		105,000				
Energy filter slit width (eV)	20		20				
Calibrated pixel size (Å/pix)	0.825		0.825				
Exposure rate (e$^-$/pix/sec)	20.97		20.21				
Frames per exposure	51		51				
Total electron exposure (e$^-$/Å^2)	55.5		53.4				
Exposure per frame (e$^-$/Å^2)	1.087		1.048				
Defocus range (µm)	-1.0, -2.3		-1.4, -2.3				
Automation software	SerialEM		SerialEM				
# of Micrographs used	22,448		27,950				
Particles extracted	2,869,130		2,325,106				
Particles after 2D classification	1,316,162		877,530				
Class	Closed	1RBD-up	Closed	1 RBD-up 1	1 RBD-up 2	1 RBD-up 3	2 RBD-up
Total # of refined particles	97,551	302,965	41,138	77,942	119,338	13,919	7,368
Symmetry imposed	C3	C1	C3	C1	C1	C1	C1
Estimated accuracy of translations/rotations	0.74/1.68	0.82/1.87	1.24/2.61	1.48/2.92	1.29/2.62	2.66/4.46	2.94/4.98
Map sharpening B-factor	-94.4	-93.1	-83.8	-90.4	-87.6	-85.3	-75.2
Unmasked Resolution at 0.5/0.143 FSC (Å)	3.88/3.38	3.73/3.19	4.17/3.47	4.45/3.77	4.21/3.57	8.80/4.89	10.42/8.08
Masked resolution at 0.5/0.143 FSC (Å)	3.53/3.11	3.3/2.9	3.70/3.14	3.84/3.33	3.70/3.21	6.94/4.00	8.08/4.30

Model refinement and validation statistics

PDB							
Composition							
Amino acids	3345	3281	3225	3258	3274	3257	3242
Glycans	57	57	57	57	57	57	57
RMSD bonds (Å)	0.013	0.013	0.013	0.013	0.013	0.013	0.015
RMSD angles (◦)	1.78	1.77	1.75	1.77	1.75	1.88	2.09
Mean B-factors							
Amino acids	97	88	99	100	99	120	135
Glycans	133	111	125	121	123	144	161
Ramachandran							
Favored (%)	93.32	93.31	93.70	93.52	93.21	91.25	90.83
Allowed(%)	5.96	6.04	6.05	5.96	6.45	8.13	8.55
Outliers(%)	0.72	0.65	0.25	0.53	0.34	0.62	0.62
Rotamer outliers (%)	0.93	1.29	1.56	0.81	0.98	1.97	1.91
Clash score	5.79	3.91	6.48	6.07	4.22	9.71	10.61
C-beta outliers (%)	0.42	0.33	0.23	0.56	0.33	0.43	0.76
CaBLAM outliers (%)	2.94	3.15	2.16	2.48	2.37	2.79	3.38
CC (mask)	0.82	0.82	0.80	0.79	0.80	0.69	0.64
CC (volume)	0.82	0.82	0.80	0.89	0.79	0.67	0.63
MolProbity score	1.75	1.70	1.92	1.76	1.64	2.25	2.28
EMRinger score	3.60	3.55	3.13	3.10	3.44	1.79	0.95

References and Notes

1. P. Zhou, X.-L. Yang, X.-G. Wang, B. Hu, L. Zhang, W. Zhang, H.-R. Si, Y. Zhu, B. Li, C.-L. Huang, H.-D. Chen, J. Chen, Y. Luo, H. Guo, R.-D. Jiang, M.-Q. Liu, Y. Chen, X.-R. Shen, X. Wang, X.-S. Zheng, K. Zhao, Q.-J. Chen, F. Deng, L.-L. Liu, B. Yan, F.-X. Zhan, Y.-Y. Wang, G.-F. Xiao, Z.-L. Shi, A pneumonia outbreak associated with a new coronavirus of probable bat origin. *Nature* **579**, 270–273 (2020). doi:10.1038/s41586-020-2012-7 Medline

2. F. Robson, K. S. Khan, T. K. Le, C. Paris, S. Demirbag, P. Barfuss, P. Rocchi, W.-L. Ng, Coronavirus RNA proofreading: Molecular basis and therapeutic targeting. *Mol. Cell* **79**, 710–727 (2020). doi:10.1016/j.molcel.2020.07.027 Medline

3. H. Tegally, E. Wilkinson, M. Giovanetti, A. Iranzadeh, V. Fonseca, J. Giandhari, D. Doolabh, S. Pillay, E. J. San, N. Msomi, K. Mlisana, A. von Gottberg, S. Walaza, M. Allam, A. Ismail, T. Mohale, A. J. Glass, S. Engelbrecht, G. Van Zyl, W. Preiser, F. Petruccione, A. Sigal, D. Hardie, G. Marais, M. Hsiao, S. Korsman, M.-A. Davies, L. Tyers, I. Mudau, D. York, C. Maslo, D. Goedhals, S. Abrahams, O. Laguda-Akingba, A. Alisoltani-Dehkordi, A. Godzik, C. K. Wibmer, B. T. Sewell, J. Lourenço, L. C. J. Alcantara, S. L. Kosakovsky Pond, S. Weaver, D. Martin, R. J. Lessells, J. N. Bhiman, C. Williamson, T. de Oliveira, Emergence and rapid spread of a new severe acute respiratory syndrome-related coronavirus 2 (SARS-CoV-2) lineage with multiple spike mutations in South Africa. medRxiv 2020.12.21.20248640 [Preprint] (2020). https://doi.org/10.1101/2020.12.21.20248640.

4. F. Grabowski, G. Preibisch, M. Kochańczyk, T. Lipniacki, SARS-CoV-2 variant of concern 202012/01 has about twofold replicative advantage and acquires concerning mutations. *Viruses* **13**, 392 (2021). doi:10.3390/v13030392 Medline

5. C. M. Voloch, R. da Silva Francisco Jr., L. G. P. de Almeida, C. C. Cardoso, O. J. Brustolini, A. L. Gerber, A. P. C. Guimarães, D. Mariani, R. M. da Costa, O. C. Ferreira Jr., A. C. Cavalcanti, T. S. Frauches, C. M. B. de Mello, I. C. Leitão, R. M. Galliez, D. S. Faffe, T. M. P. P. Castiñeiras, A. Tanuri, A. T. R. de Vasconcelos; Covid19-UFRJ Workgroup; LNCC Workgroup, Genomic characterization of a novel SARS-CoV-2 lineage from Rio de Janeiro, Brazil. *J. Virol.* **95**, e00119–e00121 (2021). doi:10.1128/JVI.00119-21 Medline

6. K. Wu, A. P. Werner, J. I. Moliva, M. Koch, A. Choi, G. B. E. Stewart-Jones, H. Bennett, S. Boyoglu-Barnum, W. Shi, B. S. Graham, A. Carfi, K. S. Corbett, R. A. Seder, D. K. Edwards, mRNA-1273 vaccine induces neutralizing antibodies against spike mutants from global SARS-CoV-2 variants. bioRxiv 2021.01.25.427948 [Preprint] (2021). https://doi.org/10.1101/2021.01.25.427948.

7. P. Wang, M. S. Nair, L. Liu, S. Iketani, Y. Luo, Y. Guo, M. Wang, J. Yu, B. Zhang, P. D. Kwong, B. S. Graham, J. R. Mascola, J. Y. Chang, M. T. Yin, M. Sobieszczyk, C. A. Kyratsous, L. Shapiro, Z. Sheng, Y. Huang, D. D. Ho, Antibody resistance of SARS-CoV-2 variants B.1.351 and B.1.1.7. *Nature* **539**, 130–135 (2021). doi:10.1038/s41586-021-03398-2 Medline

8. C. K. Wibmer, F. Ayres, T. Hermanus, M. Madzivhandila, P. Kgagudi, B. Oosthuysen, B. E. Lambson, T. de Oliveira, M. Vermeulen, K. van der Berg, T. Rossouw, M. Boswell, V.

Ueckermann, S. Meiring, A. von Gottberg, C. Cohen, L. Morris, J. N. Bhiman, P. L. Moore, SARS-CoV-2 501Y.V2 escapes neutralization by South African COVID-19 donor plasma. *Nat. Med.* **27**, 622–625 (2021). doi:10.1038/s41591-021-01285-x Medline

9. N. G. Davies, C. I. Jarvis, W. J. Edmunds, N. P. Jewell, K. Diaz-Ordaz, R. H. Keogh; CMMID COVID-19 Working Group, Increased mortality in community-tested cases of SARS-CoV-2 lineage B.1.1.7. *Nature* **593**, 270–274 (2021). doi:10.1038/s41586-021-03426-1 Medline

10. R. Challen, E. Brooks-Pollock, J. M. Read, L. Dyson, K. Tsaneva-Atanasova, L. Danon, Risk of mortality in patients infected with SARS-CoV-2 variant of concern 202012/1: Matched cohort study. *BMJ* **372**, n579 (2021). Medline

11. B. J. Bosch, R. van der Zee, C. A. de Haan, P. J. Rottier, The coronavirus spike protein is a class I virus fusion protein: Structural and functional characterization of the fusion core complex. *J. Virol.* **77**, 8801–8811 (2003). doi:10.1128/JVI.77.16.8801-8811.2003 Medline

12. M. Hoffmann, H. Kleine-Weber, S. Schroeder, N. Krüger, T. Herrler, S. Erichsen, T. S. Schiergens, G. Herrler, N.-H. Wu, A. Nitsche, M. A. Müller, C. Drosten, S. Pöhlmann, SARS-CoV-2 cell entry depends on ACE2 and TMPRSS2 and is blocked by a clinically proven protease inhibitor. *Cell* **181**, 271–280.e8 (2020). doi:10.1016/j.cell.2020.02.052 Medline

13. J. K. Millet, G. R. Whittaker, Host cell entry of Middle East respiratory syndrome coronavirus after two-step, furin-mediated activation of the spike protein. *Proc. Natl. Acad. Sci. U.S.A.* **111**, 15214–15219 (2014). doi:10.1073/pnas.1407087111 Medline

14. M. A. Tortorici, D. Veesler, Structural insights into coronavirus entry. *Adv. Virus Res.* **105**, 93–116 (2019). doi:10.1016/bs.aivir.2019.08.002 Medline

15. D. Wrapp, N. Wang, K. S. Corbett, J. A. Goldsmith, C.-L. Hsieh, O. Abiona, B. S. Graham, J. S. McLellan, Cryo-EM structure of the 2019-nCoV spike in the prefusion conformation. *Science* **367**, 1260–1263 (2020). doi:10.1126/science.abb2507 Medline

16. A. C. Walls, Y.-J. Park, M. A. Tortorici, A. Wall, A. T. McGuire, D. Veesler, Structure, function, and antigenicity of the SARS-CoV-2 spike glycoprotein. *Cell* **181**, 281–292.e6 (2020). doi:10.1016/j.cell.2020.02.058 Medline

17. J. Lan, J. Ge, J. Yu, S. Shan, H. Zhou, S. Fan, Q. Zhang, X. Shi, Q. Wang, L. Zhang, X. Wang, Structure of the SARS-CoV-2 spike receptor-binding domain bound to the ACE2 receptor. *Nature* **581**, 215–220 (2020). doi:10.1038/s41586-020-2180-5 Medline

18. R. Yan, Y. Zhang, Y. Li, L. Xia, Y. Guo, Q. Zhou, Structural basis for the recognition of SARS-CoV-2 by full-length human ACE2. *Science* **367**, 1444–1448 (2020). doi:10.1126/science.abb2762 Medline

19. J. Shang, G. Ye, K. Shi, Y. Wan, C. Luo, H. Aihara, Q. Geng, A. Auerbach, F. Li, Structural basis of receptor recognition by SARS-CoV-2. *Nature* **581**, 221–224 (2020). doi:10.1038/s41586-020-2179-y Medline

20. Q. Wang, Y. Zhang, L. Wu, S. Niu, C. Song, Z. Zhang, G. Lu, C. Qiao, Y. Hu, K.-Y. Yuen, Q. Wang, H. Zhou, J. Yan, J. Qi, Structural and functional basis of SARS-CoV-2 entry

by using human ACE2. *Cell* **181**, 894–904.e9 (2020). doi:10.1016/j.cell.2020.03.045 Medline

21. S. Xia, M. Liu, C. Wang, W. Xu, Q. Lan, S. Feng, F. Qi, L. Bao, L. Du, S. Liu, C. Qin, F. Sun, Z. Shi, Y. Zhu, S. Jiang, L. Lu, Inhibition of SARS-CoV-2 (previously 2019-nCoV) infection by a highly potent pan-coronavirus fusion inhibitor targeting its spike protein that harbors a high capacity to mediate membrane fusion. *Cell Res.* **30**, 343–355 (2020). doi:10.1038/s41422-020-0305-x Medline

22. Y. Cai, J. Zhang, T. Xiao, H. Peng, S. M. Sterling, R. M. Walsh Jr., S. Rawson, S. Rits-Volloch, B. Chen, Distinct conformational states of SARS-CoV-2 spike protein. *Science* **369**, 1586–1592 (2020). doi:10.1126/science.abd4251 Medline

23. S. Bangaru, G. Ozorowski, H. L. Turner, A. Antanasijevic, D. Huang, X. Wang, J. L. Torres, J. K. Diedrich, J.-H. Tian, A. D. Portnoff, N. Patel, M. J. Massare, J. R. Yates 3rd, D. Nemazee, J. C. Paulson, G. Glenn, G. Smith, A. B. Ward, Structural analysis of full-length SARS-CoV-2 spike protein from an advanced vaccine candidate. *Science* **370**, 1089–1094 (2020). doi:10.1126/science.abe1502 Medline

24. B. Turoňová, M. Sikora, C. Schürmann, W. J. H. Hagen, S. Welsch, F. E. C. Blanc, S. von Bülow, M. Gecht, K. Bagola, C. Hörner, G. van Zandbergen, J. Landry, N. T. D. de Azevedo, S. Mosalaganti, A. Schwarz, R. Covino, M. D. Mühlebach, G. Hummer, J. Krijnse Locker, M. Beck, In situ structural analysis of SARS-CoV-2 spike reveals flexibility mediated by three hinges. *Science* **370**, 203–208 (2020). doi:10.1126/science.abd5223 Medline

25. Z. Ke, J. Oton, K. Qu, M. Cortese, V. Zila, L. McKeane, T. Nakane, J. Zivanov, C. J. Neufeldt, B. Cerikan, J. M. Lu, J. Peukes, X. Xiong, H.-G. Kräusslich, S. H. W. Scheres, R. Bartenschlager, J. A. G. Briggs, Structures and distributions of SARS-CoV-2 spike proteins on intact virions. *Nature* **588**, 498–502 (2020). doi:10.1038/s41586-020-2665-2 Medline

26. H. Yao, Y. Song, Y. Chen, N. Wu, J. Xu, C. Sun, J. Zhang, T. Weng, Z. Zhang, Z. Wu, L. Cheng, D. Shi, X. Lu, J. Lei, M. Crispin, Y. Shi, L. Li, S. Li, Molecular architecture of the SARS-CoV-2 virus. *Cell* **183**, 730–738.e13 (2020). doi:10.1016/j.cell.2020.09.018 Medline

27. C. Liu, L. Mendonça, Y. Yang, Y. Gao, C. Shen, J. Liu, T. Ni, B. Ju, C. Liu, X. Tang, J. Wei, X. Ma, Y. Zhu, W. Liu, S. Xu, Y. Liu, J. Yuan, J. Wu, Z. Liu, Z. Zhang, L. Liu, P. Wang, P. Zhang, The architecture of inactivated SARS-CoV-2 with postfusion spikes revealed by cryo-EM and cryo-ET. *Structure* **28**, 1218–1224.e4 (2020). doi:10.1016/j.str.2020.10.001 Medline

28. J. Zhang, Y. Cai, T. Xiao, J. Lu, H. Peng, S. M. Sterling, R. M. Walsh Jr., S. Rits-Volloch, H. Zhu, A. N. Woosley, W. Yang, P. Sliz, B. Chen, Structural impact on SARS-CoV-2 spike protein by D614G substitution. *Science* **372**, 525–530 (2021). doi:10.1126/science.abf2303 Medline

29. L. Dai, G. F. Gao, Viral targets for vaccines against COVID-19. *Nat. Rev. Immunol.* **21**, 73–82 (2021). doi:10.1038/s41577-020-00480-0 Medline

30. M. S. Gebre, L. A. Brito, L. H. Tostanoski, D. K. Edwards, A. Carfi, D. H. Barouch, Novel approaches for vaccine development. *Cell* **184**, 1589–1603 (2021). doi:10.1016/j.cell.2021.02.030 Medline

31. L. R. Baden, H. M. El Sahly, B. Essink, K. Kotloff, S. Frey, R. Novak, D. Diemert, S. A. Spector, N. Rouphael, C. B. Creech, J. McGettigan, S. Khetan, N. Segall, J. Solis, A. Brosz, C. Fierro, H. Schwartz, K. Neuzil, L. Corey, P. Gilbert, H. Janes, D. Follmann, M. Marovich, J. Mascola, L. Polakowski, J. Ledgerwood, B. S. Graham, H. Bennett, R. Pajon, C. Knightly, B. Leav, W. Deng, H. Zhou, S. Han, M. Ivarsson, J. Miller, T. Zaks; COVE Study Group, Efficacy and safety of the mRNA-1273 SARS-CoV-2 vaccine. *N. Engl. J. Med.* **384**, 403–416 (2021). doi:10.1056/NEJMoa2035389 Medline

32. F. P. Polack, S. J. Thomas, N. Kitchin, J. Absalon, A. Gurtman, S. Lockhart, J. L. Perez, G. Pérez Marc, E. D. Moreira, C. Zerbini, R. Bailey, K. A. Swanson, S. Roychoudhury, K. Koury, P. Li, W. V. Kalina, D. Cooper, R. W. Frenck Jr., L. L. Hammitt, Ö. Türeci, H. Nell, A. Schaefer, S. Ünal, D. B. Tresnan, S. Mather, P. R. Dormitzer, U. Şahin, K. U. Jansen, W. C. Gruber; C4591001 Clinical Trial Group, Safety and efficacy of the BNT162b2 mRNA Covid-19 vaccine. *N. Engl. J. Med.* **383**, 2603–2615 (2020). doi:10.1056/NEJMoa2034577 Medline

33. S. S. Abdool Karim, T. de Oliveira, New SARS-CoV-2 variants—Clinical, public health, and vaccine implications. *N. Engl. J. Med.* **384**, 1866–1868 (2021). doi:10.1056/NEJMc2100362 Medline

34. B. Korber, W. M. Fischer, S. Gnanakaran, H. Yoon, J. Theiler, W. Abfalterer, N. Hengartner, E. E. Giorgi, T. Bhattacharya, B. Foley, K. M. Hastie, M. D. Parker, D. G. Partridge, C. M. Evans, T. M. Freeman, T. I. de Silva, C. McDanal, L. G. Perez, H. Tang, A. Moon-Walker, S. P. Whelan, C. C. LaBranche, E. O. Saphire, D. C. Montefiori, A. Angyal, R. L. Brown, L. Carrilero, L. R. Green, D. C. Groves, K. J. Johnson, A. J. Keeley, B. B. Lindsey, P. J. Parsons, M. Raza, S. Rowland-Jones, N. Smith, R. M. Tucker, D. Wang, M. D. Wyles; Sheffield COVID-19 Genomics Group, Tracking changes in SARS-CoV-2 spike: Evidence that D614G increases infectivity of the COVID-19 virus. *Cell* **182**, 812–827.e19 (2020). doi:10.1016/j.cell.2020.06.043 Medline

35. P. Tong, A. Gautam, I. Windsor, M. Travers, Y. Chen, N. Garcia, N. B. Whiteman, L. G. A. McKay, F. J. N. Lelis, S. Habibi, Y. Cai, L. J. Rennick, W. P. Duprex, K. R. McCarthy, C. L. Lavine, T. Zuo, J. Lin, A. Zuiani, J. Feldman, E. A. MacDonald, B. M. Hauser, A. Griffths, M. S. Seaman, A. G. Schmidt, B. Chen, D. Neuberg, G. Bajic, S. C. Harrison, D. R. Wesemann, Memory B cell repertoire for recognition of evolving SARS-CoV-2 spike. bioRxiv 2021.03.10.434840 [Preprint] (2021). https://doi.org/10.1101/2021.03.10.434840.

36. X. Zhu, D. Mannar, S. S. Srivastava, A. M. Berezuk, J.-P. Demers, J. W. Saville, K. Leopold, W. Li, D. S. Dimitrov, K. S. Tuttle, S. Zhou, S. Chittori, S. Subramaniam, Cryo-electron microscopy structures of the N501Y SARS-CoV-2 spike protein in complex with ACE2 and 2 potent neutralizing antibodies. *PLOS Biol.* **19**, e3001237 (2021). doi:10.1371/journal.pbio.3001237 Medline

37. S. M. Gobeil, K. Janowska, S. McDowell, K. Mansouri, R. Parks, V. Stalls, M. F. Kopp, K. Manne, K. Saunders, R. J. Edwards, B. F. Haynes, R. C. Henderson, P. Acharya, Effect

of natural mutations of SARS-CoV-2 on spike structure, conformation and antigenicity. bioRxiv 2021.03.11.435037 [Preprint] (2021). https://doi.org/10.1101/2021.03.11.435037.

38. S. H. Scheres, RELION: Implementation of a Bayesian approach to cryo-EM structure determination. *J. Struct. Biol.* **180**, 519–530 (2012). doi:10.1016/j.jsb.2012.09.006 Medline

39. A. Punjani, J. L. Rubinstein, D. J. Fleet, M. A. Brubaker, cryoSPARC: Algorithms for rapid unsupervised cryo-EM structure determination. *Nat. Methods* **14**, 290–296 (2017). doi:10.1038/nmeth.4169 Medline

40. X. Chi, R. Yan, J. Zhang, G. Zhang, Y. Zhang, M. Hao, Z. Zhang, P. Fan, Y. Dong, Y. Yang, Z. Chen, Y. Guo, J. Zhang, Y. Li, X. Song, Y. Chen, L. Xia, L. Fu, L. Hou, J. Xu, C. Yu, J. Li, Q. Zhou, W. Chen, A neutralizing human antibody binds to the N-terminal domain of the Spike protein of SARS-CoV-2. *Science* **369**, 650–655 (2020). doi:10.1126/science.abc6952 Medline

41. J. Wise, Covid-19: The E484K mutation and the risks it poses. *BMJ* **372**, n359 (2021). Medline

42. T. Xiao, J. Lu, J. Zhang, R. I. Johnson, L. G. A. McKay, N. Storm, C. L. Lavine, H. Peng, Y. Cai, S. Rits-Volloch, S. Lu, B. D. Quinlan, M. Farzan, M. S. Seaman, A. Griffiths, B. Chen, A trimeric human angiotensin-converting enzyme 2 as an anti-SARS-CoV-2 agent. *Nat. Struct. Mol. Biol.* **28**, 202–209 (2021). doi:10.1038/s41594-020-00549-3 Medline

43. J. Chen, J. M. Kovacs, H. Peng, S. Rits-Volloch, J. Lu, D. Park, E. Zablowsky, M. S. Seaman, B. Chen, Effect of the cytoplasmic domain on antigenic characteristics of HIV-1 envelope glycoprotein. *Science* **349**, 191–195 (2015). doi:10.1126/science.aaa9804 Medline

44. D. N. Mastronarde, Automated electron microscope tomography using robust prediction of specimen movements. *J. Struct. Biol.* **152**, 36–51 (2005). doi:10.1016/j.jsb.2005.07.007 Medline

45. S. Q. Zheng, E. Palovcak, J.-P. Armache, K. A. Verba, Y. Cheng, D. A. Agard, MotionCor2: Anisotropic correction of beam-induced motion for improved cryo-electron microscopy. *Nat. Methods* **14**, 331–332 (2017). doi:10.1038/nmeth.4193 Medline

46. A. Rohou, N. Grigorieff, CTFFIND4: Fast and accurate defocus estimation from electron micrographs. *J. Struct. Biol.* **192**, 216–221 (2015). doi:10.1016/j.jsb.2015.08.008 Medline

47. T. Wagner, F. Merino, M. Stabrin, T. Moriya, C. Antoni, A. Apelbaum, P. Hagel, O. Sitsel, T. Raisch, D. Prumbaum, D. Quentin, D. Roderer, S. Tacke, B. Siebolds, E. Schubert, T. R. Shaikh, P. Lill, C. Gatsogiannis, S. Raunser, SPHIRE-crYOLO is a fast and accurate fully automated particle picker for cryo-EM. *Commun. Biol.* **2**, 218 (2019). doi:10.1038/s42003-019-0437-z Medline

48. P. Emsley, B. Lohkamp, W. G. Scott, K. Cowtan, Features and development of *Coot*. *Acta Crystallogr. D* **66**, 486–501 (2010). doi:10.1107/S0907444910007493 Medline

49. P. D. Adams, P. V. Afonine, G. Bunkóczi, V. B. Chen, I. W. Davis, N. Echols, J. J. Headd, L.-W. Hung, G. J. Kapral, R. W. Grosse-Kunstleve, A. J. McCoy, N. W. Moriarty, R. Oeffner, R. J. Read, D. C. Richardson, J. S. Richardson, T. C. Terwilliger, P. H. Zwart, *PHENIX*: A comprehensive Python-based system for macromolecular structure solution. *Acta Crystallogr. D* **66**, 213–221 (2010). doi:10.1107/S0907444909052925 Medline

50. T. I. Croll, *ISOLDE*: A physically realistic environment for model building into low-resolution electron-density maps. *Acta Crystallogr. D* **74**, 519–530 (2018). doi:10.1107/S2059798318002425 Medline

51. A. Morin, B. Eisenbraun, J. Key, P. C. Sanschagrin, M. A. Timony, M. Ottaviano, P. Sliz, Collaboration gets the most out of software. *eLife* **2**, e01456 (2013). doi:10.7554/eLife.01456 Medline
